# Methods in quantitative biology—from analysis of single-cell microscopy images to inference of predictive models for stochastic gene expression

**DOI:** 10.1088/1478-3975/adda85

**Published:** 2025-06-10

**Authors:** Luis U Aguilera, Lisa M Weber, Eric Ron, Connor R King, Kaan Öcal, Alex Popinga, Joshua Cook, Michael P May, William S Raymond, Zachary R Fox, Linda S Forero-Quintero, Jack R Forman, Alexandre David, Brian Munsky

**Affiliations:** 1 Department of Chemical and Biological Engineering, Colorado State University, Fort Collins, CO 80523, United States of America; 2 School of Biomedical Engineering, Colorado State University, Fort Collins, CO 80523, United States of America; 3 Cell and Molecular Biology Program, Colorado State University, Fort Collins, CO 80523, United States of America; 4 School of BioSciences, University of Melbourne, Parkville, Victoria 3010, Australia; 5 School of Biological Sciences, University of Auckland, Auckland CBD, Auckland 1010, New Zealand; 6 Oak Ridge National Laboratory, Oak Ridge, TN 37830, United States of America; 7 Department of Biochemistry and Molecular Genetics, University of Colorado-Anschutz Medical Campus, Aurora, CO 80045, United States of America; 8 School of Mathematics and Engineering, Front Range Community College, Fort Collins, CO 80526, United States of America

**Keywords:** stochastic gene expression, fluorescence microscopy, model inference, quantitative biology, single-cell imaging, mechanistic models

## Abstract

The field of quantitative biology (q-bio) seeks to provide precise and testable explanations for observed biological phenomena by applying mathematical and computational methods. The central goals of q-bio are to (1) systematically propose quantitative hypotheses in the form of mathematical models, (2) demonstrate that these models faithfully capture a specific essence of a biological process, and (3) correctly forecast the dynamics of the process in new, and previously untested circumstances. Achieving these goals depends on accurate analysis and incorporating informative experimental data to constrain the set of potential mathematical representations. In this introductory tutorial, we provide an overview of the state of the field and introduce some of the computational methods most commonly used in q-bio. In particular, we examine experimental techniques in single-cell imaging, computational tools to process images and extract quantitative data, various mechanistic modeling approaches used to reproduce these quantitative data, and techniques for data-driven model inference and model-driven experiment design. All topics are presented in the context of additional online resources, including open-source Python notebooks and open-ended practice problems that comprise the technical content of the annual Undergraduate Quantitative Biology Summer School (UQ-Bio).

## 1. Introduction

**Figure 1.**
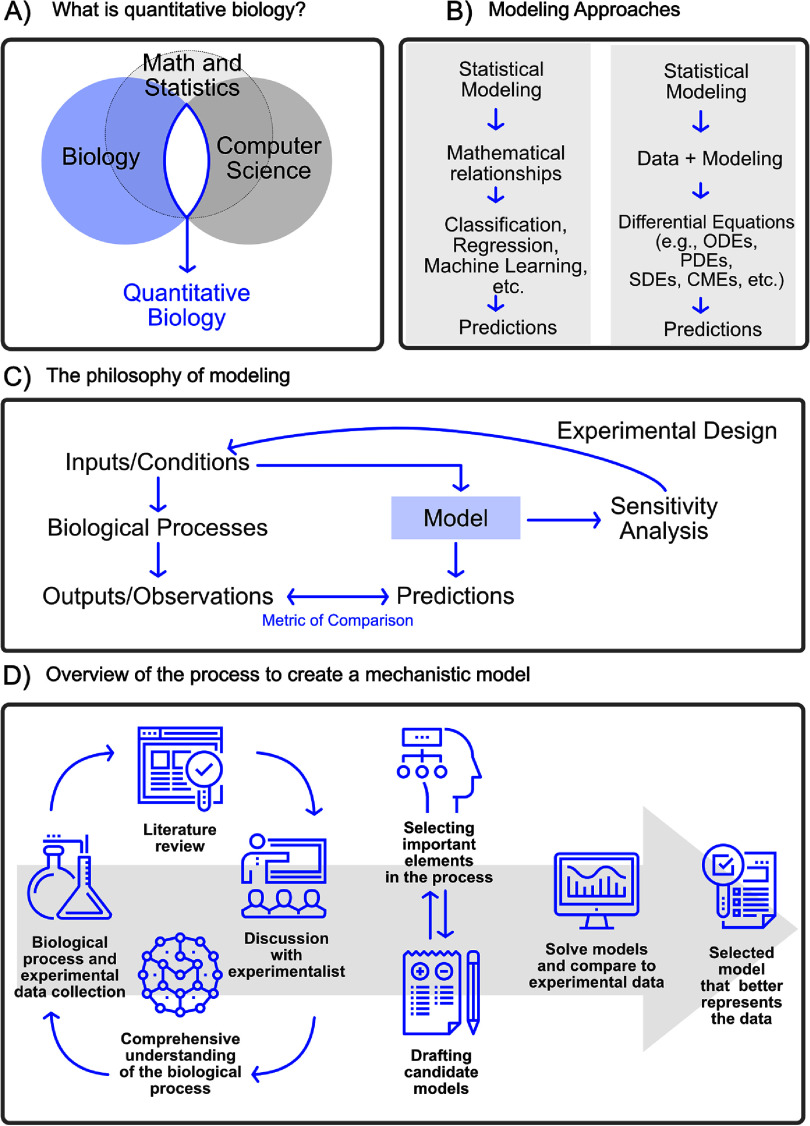
Overview of quantitative biology. (A) Quantitative biology lies at the intersection of biology, computer science, math and statistics. (B) Both statistical and mechanistic models attempt to infer relationships between experimental observations. The key difference is that mechanistic models also attempt to constrain these relationships to follow known or hypothesized physical laws or biochemical reactions. (C) Modeling involves an iterative procedure of collecting diverse data sets, making predictions for feasible experiments, constraining models to match resulting data, and analyzing model sensitivities to target more informative experimental conditions that are most likely to glean further insight. (D) The creating of mechanistic models involves close collaboration between experimentalists and computational scientists.

***Quantitative biology*** is an interdisciplinary field that integrates biology, computer science, mathematics, and engineering to understand the behavior of biological systems (figure 1(A)). By employing quantitative methods, researchers have made significant strides in studying vital biological processes, including gene expression [1], signaling pathways [2], viral dynamics [3, 4], microbial community dynamics [5], and more. Quantitative biology is crucial in advancing biomedical research [6], drug development [7], and synthetic biology [8]. The models developed in quantitative biology have far-reaching implications, ranging from designing personalized medical treatments [9] to shaping public policy during pandemics [10].

### 1.1. History of quantitative biology

Using quantitative methods to study life sciences is not new. Early examples can be traced back to the 19th century when Pierre-François Verhulst introduced the logistic function to model population growth [11]. In the late 19th century, Cato Guldberg and Peter Waage elucidated the principle for the Law of Mass Action to describe the stoichiometry and rates of elementary chemical reactions [12]. At the beginning of the 20th century, Leonor Michaelis and Maud Menten extended upon these to introduce the model describing the kinetics of substrate and products in enzyme-catalyzed reactions [13]. During the same period, Lotka–Volterra (or predator-prey) equations were introduced to model the evolution of interacting populations of distinct species [14, 15]. In the 1950s, Alan Hodgkin and Andrew Huxley introduced a mechanistic model to describe the initiation and propagation of action potentials in neurons [16]. During the ‘60s and ‘70s, scientists combined experimental observations with statistical models to determine genetic variation and predict quantitative traits in livestock and crops [17]. In 1965, Margaret Dayhoff, the ‘mother of bioinformatics,’ pioneered the development of computational methods in studying biological molecules [18]. Later in the ‘90s, large data sets of DNA, RNA, and protein sequences became available during the genome sequencing era, and the need to store and interpret these data sets gave rise to genomics and structural bioinformatics [19]. By the end of the ‘90s, important progress was made using mathematical models to understand the evolution of the immune system and HIV in infected patients, paving the way to developing potent antiviral drugs [20]. In parallel, physicists and biologists combined crystallographic data and molecular models to determine the 3D structure of proteins [21]. In the early 2000’s, systems biology was raised as a new paradigm to have a holistic (systems) understanding of all the metabolic processes in the cell [22]. At the same time, by combining mechanistic modeling and experimental data, important progress was achieved in understanding the sources of variability (extrinsic and intrinsic noise) in gene expression [23]. More recently, powerful technologies have been introduced to generate massive sets of biological data, which, combined with more powerful computers, has led to the development of complex models with predictive capabilities and a better understanding of ‘whole-cell’ processes [24]. At the same time, flux balance analyses have made significant strides to combine the concepts of mass balance and evolutionary optimization to study metabolic processes in genome scale models [25]. Nowadays, breakthroughs in biology have been achieved by using artificial intelligence and machine learning models. For example, image segmentation has been revolutionized after the introduction of U-net, a deep learning approach capable of automatically segmenting biomedical images [26]. In the realm of protein structure prediction, AlphaFold is an increasingly popular deep-learning model that has been demonstrated to predict protein structures with comparable scores to experimental results in Critical Assessment of Protein Structure Prediction competitions [27, 28]. The discovery of novel drugs is facilitated by the use of generative artificial intelligence [29].

### 1.2. History of the q-bio Conference and Summer School

As stated before, quantitative biology had been in practice for many decades before 2007, and the term ‘q-bio’ was previously coined by the physics e-print server (arXiv.org) in 2003. However, the prominence of the discipline entered a period of rapid growth following the first Annual q-bio Summer School and Conference in Cellular Information Processing (Summer 2007 at St. James College in Santa Fe, New Mexico). As Ilya Nemenman and colleagues write in their review of this inaugural event [30]:

‘[The Los Alamos based] organizers adopted the term ‘q-bio’ to succinctly refer to research efforts directed at predictive modeling of cellular regulatory systems$\ldots$ [T]he reference ‘quantitative biology’, a long-used term, recognizes that the type of work emphasized at the conference is not new, although it does seem to be reaching a new level of maturity as technological advances allow biological systems to be probed and monitored quantitatively with unprecedented control, scope, and resolution$\ldots$ The name reflects a hope that the conference will help spark a revolution that will bring the prominence of quantitative work in biology up to the level of that in fields such as chemistry and physics.’

As hoped by the original organizers, the conference certainly sparked a lasting interest in the field, and the Annual q-bio Summer School and Conference (recently celebrating their 17th year) have since hosted several thousand participants in discussions of how quantitative models can describe and predict biological processes.

### 1.3. What is this paper all about?

This review introduces some basic modeling strategies taught at the annual Undergraduate Quantitative Biology (UQ-Bio) Summer School that eventually grew from the q-bio effort. Specifically, we describe several experimental techniques that use fluorescence microscopy to visualize single-cell and single-molecule dynamics of gene expression, and we show how these data can be understood through the lenses of deterministic ODEs and discrete stochastic analyses. In section 2, we introduce the reader to a basic philosophy of quantitative modeling in biology, and we discuss some of the goals and initial strategies needed to create an appropriate model. In section 3, we review some of the modern labeling and microscopy tools used to generate single-cell images, and in section 4, we introduce the reader to some simple approaches to process these images and extract quantitative data. In section 5, we discuss modern deterministic and stochastic modeling tools used to reproduce the dynamics of single-cell processes, and section 6 presents modern tools used to integrate experimental data with these models. Due to limitations of space, we present these topic at a high level in this article, but we provide extensive and detailed online resources for the interested reader to build their skills in each topic. Specifically, the appendix provides links to comprehensive video tutorials and interactive electronic Python notebooks that demonstrate the technical material, including advice on how to choose the most appropriate modeling strategy, how to build effective procedures for managing experimental data, how to validate computational pipelines, and how to make scientific codes more accessible and reproducible. Finally, in gray boxes like the following, we describe a multipart drug discovery exercise to challenge the reader to implement all the topics discussed in this text and to build a predictive model of gene expression from simulated single-cell microscopy experiments.

### 1.4. UQ-Bio Summer School challenge—introduction

Throughout this tutorial, we will use a simulated case study to illustrate various steps toward analyzing data and creating mechanistic models to explain and predict the expression of a deleterious protein at the level of single cells. In the first stage of the challenge (section 2.3), you (the reader) will be asked to define the scope of a model based on initial knowledge about the system and available experiments. In the second stage of the challenge (section 4.4), you will be asked to process simulated microscopy videos to collect quantitative data for the spatial and temporal expression of RNA and protein at different times following application of the drug. In the third stage, you will be asked to create a mathematical framework to describe (section 5.2) and simulate the gene expression process under normal and perturbed conditions using deterministic (section 5.4) and stochastic approaches (sections 5.5 and 5.6). Finally, in the fourth stage (section 6.3), you will be asked to combine your mathematical model with the processed experimental data to quantify how well the model reproduces observed behaviors, and to infer model parameters.

This challenge is designed to increase in complexity, and readers are encouraged to complete the stages in the recommended order using Python code Jupyter Notebooks (links to example solutions are provided in each section). When this challenge has been used as part of the UQ-Bio Summer School, participating teams were asked to document and present all steps needed to formulate and simulate the model as well as to replicate the experimental data. For the sake of simplicity, here we consider a single simulated system containing one gene and one *known* drug mechanism-of-action (i.e. disruption of RNA nucleus to cytoplasm transport). In the UQ-Bio program, student teams have been asked to consider multiple drugs, each with different and *unknown* strengths and mechanisms, and they must iterate the presented steps in order to determine these mechanisms and strengths. Codes needed to generate data for these extensions are also provided in the appendix.

## 2. The basics of quantitative models

At the heart of quantitative biology is the concept of a ***model***, which we define as a mathematical representation that aims to capture a system’s essence. Quantitative biology has two general approaches to modeling biological phenomena: statistical models (in which we include machine learning) and mechanistic models (figure 1). For either type of model, the goal of the model is typically to quantitatively integrate existing knowledge (i.e. the ‘prior’) and freshly acquired data (i.e. new ‘evidence’) to predict how certain system aspects (e.g. basic properties like chemical concentration and process energy or more collective, emergent properties such as phenotype or behavior) may change (e.g. in time or space) under different experimental circumstances (e.g. under different genetic, chemical, or environmental manipulations). Although statistical and mechanistic models share similar goals, their approaches differ in their underlying mathematical principles, assumptions, and methods needed to solve them. ***Statistical models*** seek to describe probability distributions for different relationship patterns within the data. Traditional statistical methods, including linear regression, logistic regression, Bayesian inference, time series analysis, etc and more modern machine learning methods extend these traditional approaches to allow for more complicated patterns, such as decision trees, neural networks, deep learning models, support vector machines, and so on [31]. These statistical approaches are built upon a strong foundation of algebra and geometry, with deep learning taking these to the next level using GPU-enhanced computing technology.

On the other hand, ***mechanistic models*** are built on an understanding of the underlying physical or biological processes that govern the system evolution in space and time. Mechanistic models often involve physical laws and chemical principles (e.g. Newton’s laws of motion, Fick’s laws of diffusion, or Guldberg and Waage’s Law of Mass Action) that are combined with conservation of mass and energy and conveniently stated in terms of differential equations. These include continuous, deterministic models based on ordinary differential equations (ODEs) and spatial models based on partial differential equations (PDEs). To account for unknown mechanisms, these models are often extended to include random noise, giving rise to stochastic models that must be analyzed using stochastic differential equations (SDEs) and stochastic partial differential equations (SPDEs). Because many important biological processes involve discrete quanta (e.g. individual molecules, genes, cells, organisms, etc), the principles underlying ODEs, PDEs, SDEs, and SPDEs can be solved for discrete processes using finite state machines (for discrete deterministic processes), Agent-Based Models (for discrete, deterministic and spatial processes), kinetic Monte Carlo (KMC), for discrete stochastic processes) and reaction-diffusion master equation (for discrete, stochastic, and spatial processes). See the q-bio community-written textbook [32] for more detailed introduction to these methods.

In modern practice (figures 1(C) and (D)), mechanistic modeling studies almost always employ statistical analyses to estimate model parameters and mechanisms from data [33], to quantify model uncertainties [34], and to reduce models to more computationally tractable representations [35, 36]. For example, thermodynamic models employ statistical mechanics to describe ensemble behaviors while offering detailed mechanistic insights into the energy flows underlying biological processes [37]. Similarly, there is a great deal of active research to include mechanistic detail within machine learning models, leading to Physics-Informed Machine Learning models [38, 39]. These ‘grey-box’ or ‘hybrid models’ aim to get the best of both worlds—on the one hand, they overcome limitations such as incomplete mechanistic understanding, and on the other, they reduce the need for massive data sets as required by purely statistical methods [40]. The choice of modeling approach depends on many factors, including the problem at hand, data availability, and, most importantly, how the model will be used. While both statistical models and mechanistic models are indispensable in the modern study of quantitative biology, this text will focus on mechanistic models because (1) they are generally more intuitive to the novice quantitative biologist, and (2) they can often provide a more interpretable set of predictions for how a biological process of interest may respond to new and unseen genetic, chemical, or environmental conditions.

### 2.1. Why create a quantitative model?

The first step in creating any mathematical model is to decide how that model will be used: what aspects of the biological process will the model be used to capture or predict? For example, perhaps the model will be used to test different mechanistic hypotheses for how a certain transcription factor regulates ‘gene X’; perhaps it will be used to predict which other genes are overexpressed when a specific drug is used to repress expression of ‘gene Y’; or maybe the model will be used to quantify the clustering of ribosomes on the mRNA transcribed from ‘gene Z’ when a particular tRNA is depleted from the cell. Because answering each question requires a different set of knowledge or experiments, you can dramatically reduce your work by asking clear questions at the outset^9^9Important Tip—do not try to please everyone or answer every question at once! It is crucial to accept that the definition of the model scope is subjective—aspects that are critical to one group of scientists may be irrelevant to another..

The general ***aim of a model*** is to rigorously connect three aspects of scientific exploration: the ***‘mechanisms’*** that affect the process of interest, the ***‘controllable inputs’*** that can perturb the process, and the ***‘observable outputs’*** that could provide data to quantify the process.

By formalizing these three aspects in different configurations, one can make clear statements about the purpose of the model, such as:

(i) *Model-Based Hypothesis Selection*—The model will analyze the results of experiments and observations to identify important mechanisms of interest.
(ii) *Model-Generated Predictions*—The model will integrate known biological constraints to predict responses under new experimental inputs.
(iii) *Model-Guided Process Design or Control*—The model will be used to design inputs or perturbations to optimize responses under biological constraints.

### 2.2. How do I tailor my model to match my experiments and scientific questions?

To make the model more useful and to guide subsequent experimentation tasks, we must narrow the ***model scope***. For this, it often helps to draw one or more cartoons (e.g. ‘free-body diagrams’). The purpose of these cartoons is to clearly and visually define the *mechanisms* under consideration in the context of *controllable inputs* and *observable outputs* that can be examined through experimentation (see figure C2 for an example cartoon). By creating such cartoons, the scientist can target their literature research and experiment designs to focus on the most essential biological processes or modeling goals. It is best to keep experimental collaborators in the loop when creating these cartoons—not all experimental assays are equal regarding feasibility, equipment availability, accuracy, or cost.

After defining the scope of the model and the availability of prior mechanistic knowledge and potential experiment designs, the next step is to choose the correct ***model resolution***, which is the amount of detail that needs to be considered in the model. In this step, relevant elements from the known biology must be prioritized for their inclusion in the mathematical representation. What aspects have direct relevance and make unique contributions to the process dynamics, and which are more tangential or redundant? When choosing among these, it is important to note that there will be a trade-off between model complexity, the number of model parameters, and the computational resources needed to analyze these models. In other words, as the model’s complexity increases, so does the need for more computational resources to solve it correctly and more experimental resources to elucidate unknown parameters. The use of overly complex models can also lead to overfitting (fitting to noise and outliers in the data), reduced applicability, and decreased detectability of bias. Additionally, for a more robust modeling process, it is recommended to consider multiple models for different hypotheses. This way, multiple hypotheses can be tested to select those that best reproduce the experimental observations or result in the least prediction uncertainty. In the following exercise, you are asked to define the scope for a model of single-cell gene expression.

### 2.3. UQ-Bio Summer School challenge—define model scope

**Figure C1.**
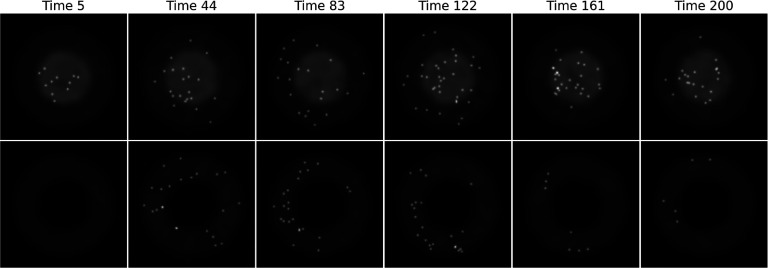
Simulated data depicting RNA and protein snapshots at different times. The figure illustrates the spatial distribution of RNA and protein concentrations within a simulated cell at six different time points. The areas enclosed by the circles represent the nucleus and the surrounding cytosol. The top row shows the RNA channel, where RNA molecules are visualized as 2D Gaussian kernels in the image. The bottom row represents the protein channel, showing the accumulation and dynamics of protein molecules synthesized from the RNA. Both rows reveal the temporal progression of gene expression, and the effects of drug-induced inhibition on mRNA transport and its subsequent impact on protein production.

The first (and often most subjective) step in any modeling endeavor is to state the goals of the modeling exercise, to identify the existing prior knowledge related to those goals, and to critically evaluate the available materials or data that are relevant to the modeling goals. In this simulated challenge, your goals are clear—you have been asked to build a model that can predict how a drug affects gene expression at the single-cell level as a function of time and drug dosage. From your experience in eukaryotic single-cell biology, you know that the gene of interest may be bursty in that it may have active and inactive transcriptional states. You know that mRNA are transcribed in the nucleus from active alleles, and that mRNA must reach the cytoplasm before they can be translated. You know that both mRNA and protein degrade over time. Moreover, you are provided with prior knowledge that the mechanism of the drug is to reduce nuclear to cytoplasmic transport of the mRNA. Finally, you expect to be provided with (simulated) microscopy images containing labeled mRNA and protein under control conditions and in response to application of the drug (see figure C1). These snapshot data have a precise spatial resolution making it possible to count individual mRNA (using smFISH) and protein (using ICC), but achieving this spatial resolution requires cell fixation, which means that it impossible to track individual mRNA or protein molecules.

Note—for the sake of simplicity, these data were simulated using a two-dimensional spatiotemporal model. To create this model, we first generated a background image using random noise, simulating the cell with two circles representing the nucleus and cytoplasm. The background was constructed for the protein and RNA channels. Transcription and translation were modeled mechanistically, using a model simulating a gene oscillating between active and inactive states. mRNA molecules are synthesized in the active state, they diffuse to the cytoplasm, and there they are translated into protein. The model is solved using the Gillespie algorithm. To integrate this model into the spatial simulation, RNA and protein molecules were represented as 2D Gaussian kernels superimposed on the simulated cytoplasm and nucleus. The movement of RNA and protein spots was modeled using a two-dimensional random walk (figure C1). The simulator is available at https://github.com/luisub/qbio_paper.git. We note that more advanced and realistic single-cell microscopy simulators are available [41, 42], but we provide this relatively simple Python script so that the reader could more easily adapt the method for their own purposes.

Armed with this information, your first task in this stage is to create a detailed schematic to represent the gene expression process within the cell. This cartoon should encompass all critical stages, such as gene activation, transcription, mRNA transport, and translation into proteins. You should label all relevant molecular species (e.g. genes, mRNA, proteins) and their interactions, and you should explicitly state what are the controllable inputs and the observable outputs.

Next, you should endeavor to select the most critical components and interactions that are essential to capture the fundamental dynamics of gene expression. When possible, try to reduce the number of species and reactions by focusing on those that significantly influence the observable system’s behavior while eliminating redundant elements that cannot be affected by the allowable perturbations or resolved from the available observations.

#### Solution—specifying a model scope

**Figure C2.**
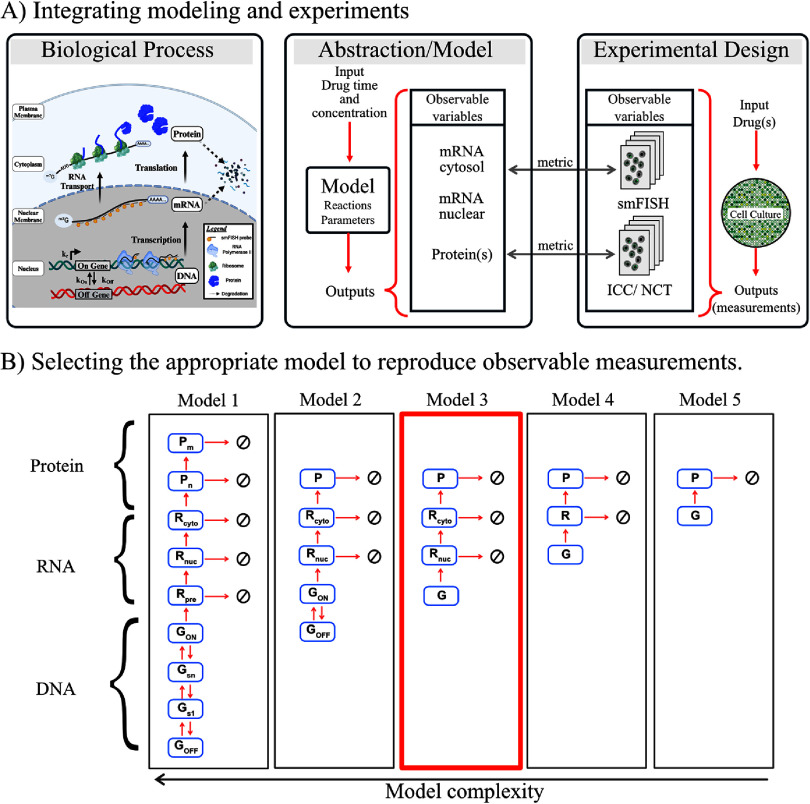
Selecting the model scope. (A) Schematic containing the steps during gene expression, including (de)activation, transcription, and translation. The center and right diagrams show how the model and experiments are integrated by comparing observable variables cytosol and nuclear mRNA measured with smFISH experiments and protein measurements with immunocytochemistry (ICC) or Nascent Chain Tracking (NTC). The direct comparison between model variables and experimental variables is achieved by using a metric described in section 6. (B) Models with different complexity. In Model 1, the diagram represents a comprehensive representation of the system including nine species; in the first part of this representation, the Gene (*G*) can transition between *n* different states until reaching an active form (*G*_*ON*_). Subsequently, multiple forms of mRNA are taken into account, including premature mRNA (*R*_*pre*_), mature nuclear mRNA (*R*_*n*_), cytoplasmic mRNA (*R*_*c*_), nascent Protein (*P*_*n*_), and mature protein (*P*_*m*_). All 16 arrows connecting the different chemical species represent the system’s transition rates (parameters). From Models 2 to 5, it can be observed that only some elements are considered in these models, reducing the complexity of the model in this way. In this example, Model 3 is selected for the rest of the exercise as it contains the minimal variables needed to reproduce the observable experimental variables. Created in BioRender. Ron E (2025). https://BioRender.com/e39n811.

To construct a mathematical representation of gene expression, we illustrate the process through a simplified diagram encompassing all the critical stages, such as gene activation, transcription, mRNA transport, and protein production. The cartoon depicted in figure C2 visualizes these gene expression phases. The models labeled 1 through 5 vary in complexity and detail. For instance, Model 1 comprises nine chemical entities and requires 16 parameters. In contrast, Model 5 simplifies to just two chemical entities with only two parameters. Moreover, this set of five models is already a major simplification; each of these models could be extended to include spatial diffusion of the genes, RNA, and protein molecules as well as to assign multiple post-transcriptional or post-translational states to control the activity and degradation of the molecules of interest. Any, all, or none of these models may be sufficient depending on the scientific question, the quality of the available experimental data, and the practicality of solving the model given the chosen methods and computational resources. As such, it is often crucial to consider multiple models.

For the purposes of this demonstration, Model 3 is selected for the rest of the exercise. The reason for this subjective choice is that Model 3 contains the minimal variables needed to capture the observable experimental variables, which are the number of mRNA in the nucleus, the number of mRNA in the cytoplasm, and the number of proteins in the cell. However, it should be noted that this choice should be flexible; if in later stages, we find that gene bursting is substantial (e.g. if the gene activation and deactivation rates are very slow compared to mRNA degradation events), then Model 3 may fail to capture large amounts of observed cell-to-cell heterogeneity, and we may need to use Models 1 or 2. Conversely, if we were to examine a drug that affects some process downstream from mRNA transport, then perhaps Model 4 or 5 may be sufficient to capture the relevant dynamics. If a different experiment were devised that allowed for direct tracking of mRNA or protein and if the specific mechanisms of diffusion were of particular interest, then a fully spatial model may be necessary. In practice, one would return to this stage later and ask critically if (1) does the chosen model capture all important phenomena (if not the model complexity may need to be increased), and (2) does the uncertainty in the model allow for acceptably precise predictions (if not, the model may need to be simplified).

## 3. Methods for single-cell analysis

Every model begins as a hypothesis that must be constrained or validated against experimental data before being trusted to describe or predict a biological phenomenon. To apply the scientific method and compare models to experiments, it is crucial to determine which experiments are needed to generate observational data, estimate errors associated with these data, and formulate quantitative metrics to rigorously compare models to data in light of these errors.

These decisions must consider the fact that different experimental techniques have vastly different powers for spatial and temporal resolution. Some techniques can measure single molecules, while others only measure average concentrations. Some techniques can measure events occurring in milliseconds, while others measure changes over long periods. Some measurements can detect the studied molecule directly, while others may use an indirect measurement such as intensity in a microscope image.

Given these differences between experimental techniques, it is necessary to determine the required spatiotemporal resolution to address the scientific question correctly and then select the appropriate modeling approach and experiment. For example, if it is known that ‘gene X’ has a large and homogeneous expression within cell populations, but it is not clear how its expression changes over time in response to chemical stimuli, then an experiment capable of measuring bulk mRNA or protein concentrations at multiple times should be sufficient to constrain a deterministic model to answer that scientific question. In contrast, if the scientific goal is to determine how the expression of ‘gene X’ is regulated, and it is known that ‘gene X’ is highly variable from one cell to the next, then experiments capable of single-molecule or single-cell resolution will be needed to constrain a stochastic model and achieve that goal. Several experimental techniques are aimed at providing bulk quantitative measurements of gene expression in terms of DNA, RNA, and proteins to satisfy the needs of the former example. These techniques include PCR, qRT-PCR, ELISA, and western blots, among others. However, in this article, we are particularly interested in gene regulation processes that lead to single-cell variations, and we will focus on single-cell experiments for the remainder of this tutorial.

### 3.1. Methods for single-cell imaging

Gene expression is a central biological process encoded in the DNA, which in most animal cell lines is present at levels of only one or two gene alleles per cell. DNA alleles fluctuate between active and inactive states. When active, genes are used as templates to produce mRNA, and these mRNAs are used as templates to produce proteins through transcription and translation processes, which in turn may also trigger signals to activate or deactivate other genes [43, 44]. In recent years, multiple methodologies have been developed to measure gene expression at single-cell and single-molecule resolution. These methods include flow cytometry [45], single-cell RNA and DNA seq [46–48], and single-cell imaging [49]. In the following, we will focus on single-molecule fluorescent microscopy; nevertheless, we remark that all other techniques can produce experimental data that can be integrated with mechanistic models using similar principles.

### 3.2. Fluorescent labels

Various fluorescent labels and activatable probes have been developed over the years to study biochemical processes at the molecular level in living and fixed cells. Most rely on combining three technologies: fluorescent molecules, methods to physically link the fluorescent molecules to a region or molecule of interest, and fluorescent microscopy. Fluorescence occurs when a fluorophore (e.g. a chemical dye molecule or bio-luminescent protein) absorbs light energy at one wavelength and then re-emits some of that energy at another lower energy wavelength [50]. Some examples of chemical fluorophores include Rhodamine [51], Alexa Fluor Dyes [52], Cyanine Dyes (Cy3, Cy5, and Cy7) [53]. It is also commonplace to utilize natural fluorophores like fluorescent proteins (e.g. the famous green fluorescent protein, GFP) [54].

### 3.3. Labeling techniques employed in fixed cells

Fixing and immobilizing the cell is often necessary to obtain the crispest spatially-resolved images of labeled biomolecules. Irreversibly arresting all cellular processes sacrifices temporal resolution, but it allows for applying highly specific labeling procedures, as follows. Immunolabeling allows identifying an antigen in a cell or tissue; the antigen is usually a protein, and full antibodies are used for detection. Full antibodies cannot pass the cell membrane; as a result, this approach is only relevant to fixed and permeabilized cells (which unfortunately have the side effect of killing the cell) or the extracellular side of the membrane for live cells [55–57].

**Figure 2.**
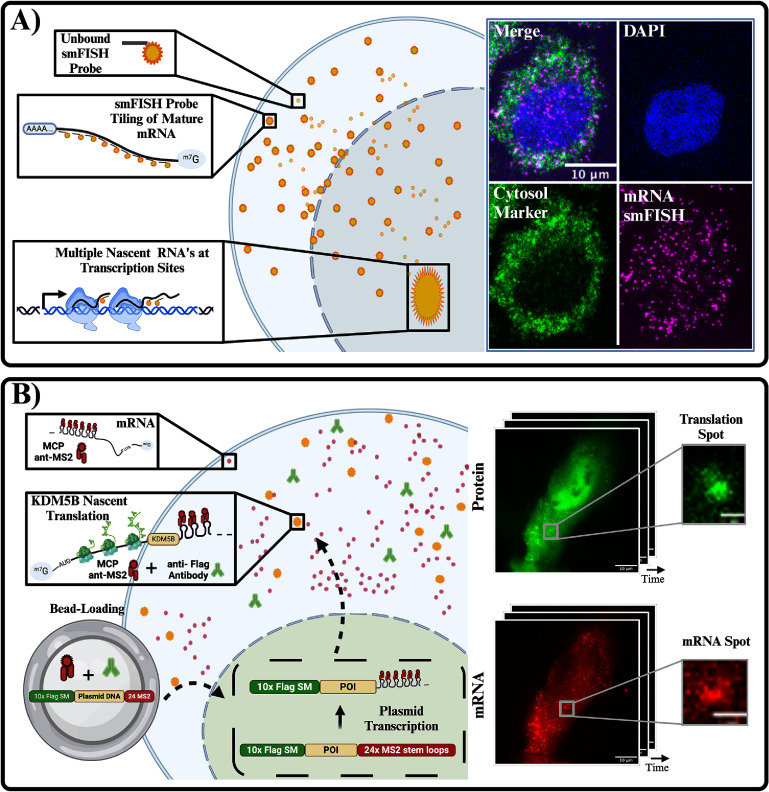
Methods in single-cell imaging. (A) Schematic representation of smFISH (single-molecule fluorescence *in situ* hybridization) for detecting mRNA in fixed cells. The top panels show unbound smiFISH probes, hybridization to mRNA via tiling, and visualization of nascent transcripts at transcription sites. Right: Confocal image showing a nucleus stained with DAPI (blue), cytosolic marker (green), and mRNA smiFISH signal (magenta) in a single cell. (B) Cartoon depicting live-cell imaging of translation using nascent chain tracking (NCT). The plasmid construct encodes the protein of interest (POI) fused to a Flag epitope tag and 24× MS2 stem loops. The plasmid is bead-loaded into cells along with fluorescent anti-Flag antibodies and MCP (MS2 coat protein). Transcription of the plasmid produces mRNA containing 24× MS2 stem loops, which bind MCP and are visualized as diffraction-limited red spots. Nascent translation of the mRNA is tracked via the binding of anti-Flag antibodies (green) to the Flag epitope on nascent chains, resulting in colocalized green and red signals (yellow). Insets show time-lapse snapshots of protein and mRNA signals. Created in BioRender. Ron E (2025). https://BioRender.com/e39n811.

Single-molecule fluorescence *in situ* hybridization (smFISH, figure 2(A)) quantifies endogenous transcription in single cells. This method targets RNA using multiple hybridized fluorescent oligonucleotides [58]. There are various types of smFISH depending on the probe design, such as (a) the original smFISH design, which is 20–50 bases long with 1–5 fluorophores per probe [59, 60], (b) indirect labeling by smiFISH consists of using multiple (20–30) primary probes, each containing the sequence targeting different regions within the gene of interest and a common sequence that is complementary to a fluorescently labeled secondary probe [61], and (c) multiplexed smFISH comprises primary probes with two sequences of readout. These are detected through successive rounds of hybridization and collectively form a code that identifies the bound cellular RNAs. Using this technique, hundreds of distinct RNA species can be detected [62–64].

### 3.4. Labeling techniques employed to visualize gene expression in live cells

Live-cell imaging enables the real-time visualization of transcription and translation dynamics in live cells. These techniques consist of encoding secondary structures alongside the biomolecule of interest and detecting them with fluorescent tags. Techniques for live visualization of transcription responses include MS2 [65] and PP7 tagging-systems [66], which are naturally occurring stem-loop structures that are recognized by bacteriophage coat proteins tagged with fluorophores.

More recent techniques have also allowed for the live-cell imaging of translation dynamics. The most prominent example is Nascent Chain Tracking (NCT, figure 2(B)). NCT employs the MS2-MCP system to label RNA by inserting the stem-loops in the 3′ untranslated region to allow for the visualization of the RNA and repeat epitope tags are encoded into the gene of interest to visualize nascent peptides [67]. Fluorescent probes bind to these repeated epitopes in this technique, illuminating the nascent protein as it is being translated [49, 68]. Recent progress using this technique includes the use of genetically encoded antibody-based probes, such as MoonTag nanobody [69], anti-HA Frankenbody [70], multiple frame tags, such as the MASH tag [69], and the multi-frame tag that allows lit-up translation in different colors depending on what frame is being translated [71, 72]. See the appendix for a link to an online video tutorial detailing these methodologies and providing several example images and videos.

## 4. Methods for processing single-cell images

Using the labeling approaches from the previous section, the images or videos captured by a fluorescence microscope contain essential data to understand single-cell dynamics. For example, one could extract data to quantify and characterize the expression of RNA or protein over time or space, either within one cell or from one cell to the next, or one could analyze changes in cell morphology after a given treatment. This section introduces fundamental concepts and approaches to process microscopy images and extract such quantitative data.

### 4.1. Representing images as data arrays

A ***microscope image***, depicted as a digital image (figure 2, Middle), is essentially a mathematical function denoted as $f(x,y)$, where *x* and *y* represent positive integer values corresponding to positions in a 2D coordinate system, specifically, the pixels [73]. To understand how information is stored in a digital image, consider it as an array of data; for example, a 2D black and white image is a matrix, a two-dimensional array with rows and columns. The matrix notation **B**_*y*,*x*_ will be used for simplicity. A more complex example could be a color image that can be represented as a three-dimensional array of data or a tensor with a shape of $\textbf{B}_{y,x,c}$, where the *c* represents a dimension containing the different color channels in the image. A color image usually comprises three channels in the order RGB (red, green, and blue). Still, it is important to note that this order is arbitrary, and other conventions exist. More generally, a ***video*** is a sequence of images taken at multiple frames. A video is also a higher-order tensor with a shape $\textbf{B}_{y,x,c,f}$, where *f* represents a dimension storing all the frames in the video. More complex stacks of images exist; for example, a sequence of microscope images with 3D spatial information (*xyz*) will result in a tensor with the shape $\textbf{B}_{z,y,x,c,f}$.

Temporal, spatial, and intensity resolution define the amount of information stored in a microscope image or video. ***Temporal resolution*** indicates the frame rate of the sequence of images. Frame rate values are typically given in units of Hertz (s^−1^). ***Spatial resolution*** describes the physical distance each pixel represents for the real object in the image. ***Intensity resolution*** represents a range of values each pixel can take in the image. This is also known as the bit-depth and is defined as 2^*n*^, where *n* is the number of bits. For example, *n* = 1, represents a binary image with values 0 and 1, *n* = 8 is an 8-bit image with a range of 0 to 255, and *n* = 16 is a 16-bit image with a range of 0 to 65 535.

### 4.2. Processing images to focus on pertinent details

Image processing involves applying mathematical operations to the image to extract relevant information. Image processing can be divided into two main areas: image warping and filtering. ***Image warping*** consists of changing the domain or pixel location in the image via a coordinate transformation. Two common examples of image warping are image registration (e.g. rotating, shifting, scaling, or shearing one image to match the coordinates of another) and data augmentation (e.g. interpolating over an image to produce a higher-resolution image). The second class of manipulation is known as ***filtering***, and these operations consist of applying logical operations, algebraic manipulations, or convolutions to adjust the intensity values of the image, often to highlight specific features within the image [73].

**Figure 3.**
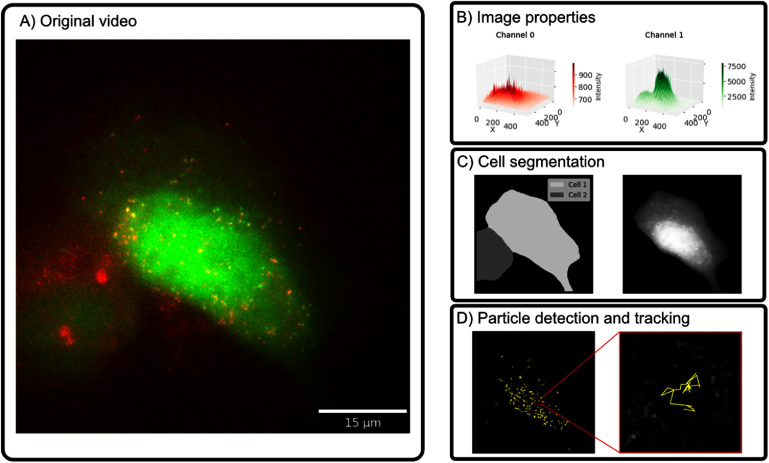
Processing fluorescence microscopy images and videos. (A) Microscope image that shows an NCT experiment that detects KDM5B RNA molecules in the red channel and nascent proteins in the green channel (see figure 2(B)). (B) Each pixel in a digital image contains an intensity value, representing the properties in the original image. Spots are observed as intensity ‘spikes’, and structures such as the nucleus can be observed as extensive elevations (3D representation showing the intensity values as the *z*-axis). (C) Cell segmentation involves detecting and labeling the regions corresponding to independent cells. Here, cell segmentation was performed using Cellpose [74] in the green channel. (D) Detected RNA spots were detected for all time frames in the original video; after this, particle trajectories were created by linking particle positions at multiple time points. Here, spot detection and tracking were performed using TrackPy [75].

There are multiple instances where such transformations are used to adjust the intensity values of an image, with a couple of the most notable including thresholding and filtering. ***Thresholding***, which is often used to remove extreme values in the image, is achieved by defining an intensity threshold value, *I*_*ts*_, based on the image’s intensity histogram (figure 3(B)) and by reducing the intensity range as follows: $\textbf{B}[\textbf{B} > I_{ts}] = I_{ts}$. Thresholding can also segment elements in the image by removing the pixels below a threshold value considered the ‘background’ in the image. For example, a simple segmentation can be achieved by binarizing the intensity values in the image. This is done by setting pixels with values below the threshold as zero and the pixels with values above the threshold to one, that is $\textbf{B}[\textbf{B} < I_{ts}] = 0$ and $\textbf{B}[\textbf{B} > = I_{ts}] = 1$.

Applying ***convolutional filters*** to images is a routine process intended to reduce noise or enhance the appearance of elements in the image. Filters are convolutions between a kernel matrix and the original image applied at a given stride. The definition of the kernel matrix is problem-specific for tasks such as noise reduction, edge detection, particle detection, noise-induced feature enhancement, etc. A comprehensive list of these matrices is available in Python using the scientific library Scikit-image [76]. For example, one of the most common filters, the ***Gaussian filter***, consists of multiplying a 2D-Gaussian square kernel matrix with a size of *k* (a user-defined parameter that normally ranges from 3 to 7 pixels) to each element in the image. A Gaussian filter reduces noise in the image by averaging adjacent pixels in a given area.

### 4.3. Extracting cells and sub-cellular features from microscopy images

In most cases, only a small portion of a given microscopy image or video is relevant, and one needs to focus attention on the specific features of the image that relate to the biological question of interest. Two of the most common feature quantification tasks in single-cell research are cell segmentation and particle detection.

***Cell segmentation*** consists of locating the pixels containing cells in a microscope image and is usually one of the first steps in any image processing pipeline for single-cell biology. Previously, cell segmentation was a time-consuming and labor-intensive manual process in which many researchers resorted to drawing cell outlines by hand. Nowadays, sophisticated image-processing libraries have been introduced recently to automate this procedure. For example, Cellpose, Detectron2, and Segment Anything Model (SAM) are deep-learning libraries for cell segmentation [74, 77, 78]. These libraries have been proven to achieve state-of-the-art segmentation of cells in microscope images. Deep-learning approaches are highly relevant to automating and reducing user input during the process of large datasets [26]. A nuclear segmentation in our example image is shown in figure 3(C).

***Particle detection*** consists of finding and quantifying punctae (bright spots), such as those corresponding to single mRNA in smFISH [60] or live-cell translation experiments [67]. One example library for spot detection and particle tracking is TrackPy [75], which contains many methods to detect punctae in the image, and more algorithms [79, 80]. Additionally, it contains robust methods to link trajectories and calculate their displacement. More specific libraries, such as Big-FISH [81], have also been introduced to detect spots in images with complex spot composition. Single punctae representing transcription sites are detected in our exemplary image using TrackPy in figure 3(D).

For more information on image processing of microscope images, the appendix provides links to tutorial notebooks and lecture videos introducing image processing basics, cell segmentation, and particle tracking. The following exercise suggests a hands-on exercise in which the reader can practice these skills.

### 4.4. UQ-Bio Summer School challenge—processing fluorescence microscopy images

The goal of the next stage of the challenge is to process the collected single-cell fluorescence microscopy images. First, you will need to create and implement a computational method to segment the cytosol and nucleus for each cell in the simulated images. For this, you should consider utilizing manual traditional techniques, such as applying combinations of filters and thresholds or by applying more automated machine learning-based segmentation routines (e.g. using Cellpose [74] or Detectron2 [77]).

Once you have segmented your images into nuclei and cytoplasm, apply particle detection algorithms to identify and locate RNA and protein spots within both the cytosol and nucleus across each color channel. Once again, traditional techniques, such as taking the differences of Gaussian filters, followed by thresholds can quickly find many spots, while programs like TrackPy [75] can be employed to detect particles based on predefined parameters such as diameter, intensity, and minimum mass. In either case, you will need to visually inspect your results to ensure that the detection parameters are optimized to accurately capture the relevant molecules without excessive false positives or negatives.

After detecting your spots, you will need to assign each detected spot to its corresponding cellular compartment (cytosol or nucleus) based on its position relative to the segmentation masks. Count the number of RNA and protein molecules detected in each compartment for every time point. Ensure that the counts are organized in a consistent structured format (e.g. use Python library Pandas Dataframes) as you are going to need to make use of these data in subsequent stages of the challenge. Write additional Python functions to generate visual representations (e.g. histograms, scatter plots, or heatmaps) to illustrate the distribution and concentration of molecules within each compartment over time.

#### Solution—processing fluorescence images

**Figure C3.**
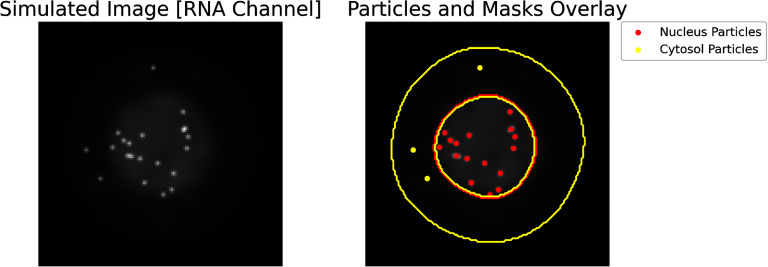
Cell segmentation and spot detection. Representative image illustrating cell segmentation and particle detection in the RNA channel. The left panel displays the original simulated RNA channel image after Gaussian smoothing. The right panel overlays the detected particles and segmentation masks onto the RNA channel image. Inner yellow contour delineates the segmented nucleus, while outside yellow contours outline the segmented cytosol. Detected RNA particles within the nucleus are marked in red, and those within the cytosol are marked in yellow.

**Figure C4.**
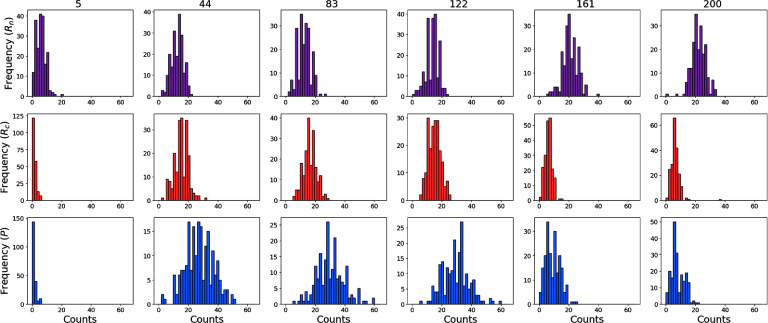
Histograms of processed data. Histograms illustrating the distributions of recovered counts for the simulated data across six time points. Top panels display the distribution of nuclear mRNA molecules. The middle panels show the distribution of cytosolic mRNA molecules. The bottom panels illustrate the distribution of protein molecules in the cytosol. Each histogram is constructed with 25 bins ranging from 0 to 65 particles.

To segment the simulated images, we used a multi-level thresholding approach. First, the simulated image was smoothed using a Gaussian filter to reduce noise. Then, the smoothed image was segmented using the Multi-Otsu thresholding algorithm [82] into three regions, including background, cytosol, and nucleus. Masks for the nucleus and cytosol were extracted, and the cytosol mask was expanded using a binary dilation method to ensure complete coverage. mRNA and protein particles were detected using TrackPy [75], using a particle size of 5 pixels, and a threshold of 20 intensity units. Detected particles were assigned to specific regions based on their positions relative to the segmentation masks (figure C3). The final counts were reported as the number of spots detected in each RNA and protein channel localization within different cellular compartments for each time and are shown as histograms in figure C4.

## 5. Quantitative mechanistic modeling

In this section, we introduce the mathematical formalism needed to represent and simulate a biological system. We start by introducing the concepts of stoichiometry and propensity functions in section 5.1. Then, we demonstrate how these can be used to simulate the system behavior in either a deterministic setting in section 5.3.1 or using a stochastic description in sections 5.4.1 and 5.5.1. Although we focus on building a quantitative description of gene expression, these same approaches can be used to describe many other biological phenomena.

### 5.1. Stoichiometry and propensity functions

To mathematically characterize a biological system, it is necessary to fully describe the system in terms of the different chemical species within it, the initial values of these species, the rules and probabilities that govern the interaction between these species, and the total time the system will be simulated. To simulate the system, the approximation assumption that the system is well-mixed and has a continuous time evolution is often made. If the latter is true, the system follows ***Markovian dynamics***, where each state transition is random, discrete, and depends only upon the system’s current state.

To define the elements (species) that form our system, we introduce the ***population vector***, $\textbf{x} = [\xi_1, \xi_2, \ldots, \xi_N]^T$, where *ξ*_1_ through *ξ*_*N*_ correspond to each of the species in the system. A particular state of the system, *i*, is denoted by the vector **x**_*i*_, which stores the count for every species in the system; hence, it contains one non-negative integer for each species.

To characterize *how* the species in the system change, we define the ***stoichiometry matrix***, **S**, with one row for each of the *N* species and one column for each of the *M* reactions, which indicates the relative (net) change in the population of each species if that reaction occurs. The stoichiometry matrix is defined as $ \textbf{S} = [\textbf{s}_{1},\textbf{s}_{2},\ldots,\textbf{s}_{\mu}]$, where $\textbf{s}_{\mu}$ is the stoichiometry column vector associated with the $\mu\textrm{th}$ reaction. When the $\mu\textrm{th}$ reaction occurs, the system transitions from it current state, **x**_*j*_, to a new state, $\textbf{x}_i = \textbf{x}_j + \textbf{s}_{\mu}$. For example, if the $\mu\textrm{th}$ hypothetical chemical reaction in a three-species system consumes two molecules of species 1 and one molecule of species 2 and produces three molecules of species 3, then the stoichiometry vector of that reaction is $\mathbf{s}_\mu = [-2,-1,3]^\textrm{T}$.

To quantify *when* the species in the system change, we introduce the ***propensity function***, *w*, as the rate (or transition probability) associated with each state transition. The propensity function is always non-negative and is a function that typically depends upon the current reactant populations and time. The term $w_{\mu}(\textbf{x},t) dt$ is the probability that the $\mu\textrm{th}$ reaction, initially in state **x**, will occur in the time-step *dt*. In many models, the law of mass action (LMA) can be applied to determine propensities for individual reactions, which states that the rate of a chemical reaction is proportional to the number of unique combinations by which those reactants can form products [83]. For the previous example, the LMA would suggest a propensity function of $w_\mu = k_\mu \left(\begin{matrix}\xi_1\\2\end{matrix}\right)\left(\begin{matrix}\xi_2\\1\end{matrix}\right)$, where *k*_*µ*_ is a rate parameter, and the quantity $\binom{\xi_1}{2}\binom{\xi_2}{1} = \left(\frac{\xi_1(\xi_1 - 1)}{2}\right)\left(\frac{\xi_2}{1}\right)$ is the number of unique molecule triplets containing two molecules of species one and one molecule of species two. See the appendix for links to tutorial videos and Python notebooks containing more details on how to specify stoichiometry vectors and propensity functions for LMA and more complex models, and see the following example to practice the skill of defining such models.

### 5.2. UQ-Bio Summer School challenge—specifying stoichiometries and propensities for a gene regulation model

In the next stage of the course challenge, you are asked to build a mathematical framework to define and then simulate a quantitative representation of the mechanisms you specified in the first stage of the challenge.

First, you should make a list of all species in the model (i.e. active/inactive alleles, nuclear mRNA, cytoplasmic mRNA, protein) and possible reactions that can occur to change these populations. Then, for each reaction, you should write out the stoichiometry vector for that reaction (i.e. how the reaction causes the populations of each species to change) and the propensity function (i.e. the formula by which the rate of that reaction changes with the populations of all species). When possible, for each reaction rate or other relevant parameter within your propensity functions, specify the expected range of that parameter to ensure they are consistent with the biological process or literature values. Organize your model in the format of a stoichiometry matrix, a propensity function vector, and a parameter table list that will be suitable for subsequent computational simulations.

#### Solution—stoichiometry and propensity functions

To model gene expression, we can use the constitutive model (Model 3 from figure C2), in which the gene is transcriptionally active and produces nuclear mRNA molecule, *R_n_*, through transcription. Nuclear RNA is transported from the nucleus to the cytosol ($R_n \rightarrow R_c$), where mRNA molecules in the cytosol can produce a protein molecule through translation. mRNA molecules and proteins are subject to degradation. Assuming that the gene concentration in the system is constant (i.e. there is a single allele and it is always active), we can define the following variables:

**Table C1.** Model variables.

Variable	Description
*R*_*n*_	mRNA nuclear
*R*_*c*_	mRNA cytoplasm
*P*	Protein

The reactions for this gene expression model are:

**Table C2.** Model reactions.

Index	Reaction	Description	Reaction Rate
*r*_1_	${\phi} \xrightarrow[]{k_{r}} {R_{n}}$	Constitutive production of nuclear mRNA	*k*_*r*_
*r*_2_	$R_{n} \xrightarrow[]{k_{t}} {R_{c}}$	mRNA transport to cytoplasm	$k_{t} \cdot [R_{n}]$
*r*_3_	${R_{c}} \xrightarrow[]{k_{p}} {R_{c} + P}$	mRNA in cytoplasm produces Protein	$k_{p} \cdot [R_{c}] $
*r*_4_	${R_{n}} \xrightarrow[]{\gamma_{r}} {\phi}$	Nuclear mRNA decay	$\gamma_{r} \cdot [R_{n}] $
*r*_5_	${R_{c}} \xrightarrow[]{\gamma_{r}} {\phi}$	Cytoplasm mRNA decay	$\gamma_{r} \cdot [R_{c}] $
*r*_6_	${P} \xrightarrow[]{\gamma_{p}} {\phi}$	Protein decay	$\gamma_{p} \cdot [P]$

with a population vector of $\textbf{x} = [R_{n}, R_{c},P]^T$. The stoichiometry matrix is:

\begin{align*} &amp;~~~~~~~~~\begin{matrix} r_{1} &amp; r_{2} &amp;\; r_{3} &amp;\; r_{4} &amp;\; r_{5} &amp;\;\;\; r_{6} \\ \end{matrix}\nonumber\\ &amp;\mathbf{S} = \begin{bmatrix} 1 &amp; -1 &amp; 0 &amp; -1 &amp; 0 &amp; 0 \\ 0 &amp; 1 &amp; 0 &amp; 0 &amp; -1 &amp; 0 \\ 0 &amp; 0 &amp; 1 &amp; 0 &amp; 0 &amp; -1 \\ \end{bmatrix}.\end{align*}

Note that the columns correspond to the stoichiometric coefficients for the reactions *r*_1_ through *r*_6_, and the rows to the species in the **x** vector. The propensity vector for this example is

\begin{equation*} \mathbf{W} = \left[ k_{r}, k_{t} R_{n}, k_{p} R_{c}, \gamma_{r} R_{n}, \gamma_{r} R_{C} , \gamma_{p} P\right]^T.\end{equation*}

### 5.3. Mechanistic simulation approaches

#### 5.3.1. Deterministic modeling approach

To describe system dynamics in a bulk setting where all species populations are assumed to be large, one can use a system of coupled ODEs. In this deterministic description, each ODE in the system describes the mean behavior over time for a specific species population. The ODE description of a system is given in terms of the stoichiometry vectors and propensity functions according to:

\begin{align*}\frac{\mathrm{d}\textbf{x}}{\mathrm{d}t}&amp;= \sum_{\mu = 1}^{M}\mathbf{s}_{\mu} w_{\mu}\left(\textbf{x}\right) = \mathbf{s}_{1}w_{1}\left(\textbf{x}\right)+ \mathbf{s}_{2} w_{2}\left(\textbf{x}\right)+ \cdots + \mathbf{s}_{M} w_{M}\left(\textbf{x}\right) = \mathbf{S}\mathbf{W},\end{align*}

where **S** is the stoichiometry matrix defined above, and $\textbf{W} = [w_1(\mathbf{x},t),\ldots,w_{M}(\mathbf{x},t)]^\textrm{T}$ is the propensity vector representing the propensity of each reaction in the system. This resulting system of ODEs can be easily solved using an ODE solver, such as those available in scipy.integrate in Python. See the appendix for additional resources and lecture videos, and complete the following example to practice building ODE analyses for gene regulation models.

### 5.4. UQ-Bio Summer School challenge—deterministic simulation of gene regulation

Now that you have defined the stoichiometry matrix, propensity function vector, parameters and initial conditions (section 5.2), use these to derive a system of ODEs to describe the rate of change of each species over time. Define initial concentrations and parameter values based on biological data or literature sources. Solve for the steady-state concentrations of each molecular species by setting the rate of change to zero and solving the resulting algebraic equations. Use computational tools (e.g. Python’s scipy.integrate.odeint) to numerically solve the ODEs and obtain time-course data for each species. Generate plots to illustrate the temporal dynamics of gene expression, and determine key behaviors such as steady states and responses to perturbations.

#### Solution—ODE representation of model

If we let $\mathbf{x} = [R_n,R_c,P]$ denote the concentrations of the three species in our model, we can now use the stoichiometric matrix and propensity function vector to directly state the ODE that describes the process as:

\begin{equation*} \frac{\mathrm{d} \mathbf{x}}{\mathrm{d}t} = \mathbf{S}\mathbf{W}.\end{equation*}

By expanding the matrix vector algebra on the right hand side of this equation, we get:

\begin{align*} \frac{\mathrm{d}\left[R_{n}\right]}{\mathrm{d}t} &amp; = k_{r} - k_{t} \cdot \left[R_{n}\right] - \gamma_{r} \cdot \left[R_{n}\right],\end{align*}

\begin{align*} \frac{\mathrm{d}\left[R_{c}\right]}{\mathrm{d}t} &amp; = k_{t} \cdot \left[R_{n}\right] - \gamma_{r} \cdot \left[R_{c}\right],\end{align*}

\begin{align*} \frac{\mathrm{d}\left[P\right]}{\mathrm{d}t} &amp; = k_{p} \cdot \left[R_{c}\right] - \gamma_{p} \cdot \left[P\right].\end{align*}

**System’s steady state**. The analytical solution for our system at steady state can be found by setting the net change in our system equal to zero. With this, we can obtain the solution for all variables in the system as a function of the parameters. That is:

\begin{equation*} R_{n}^* = \frac{k_{r}}{\gamma_{r} + k_{t}},\end{equation*}

\begin{equation*} R_{c}^* = \frac{k_{t} \cdot R_{n}^*}{\gamma_{r}},\end{equation*}

\begin{equation*} P^* = \frac{k_{p} \cdot R_{c}^*}{\gamma_{p}}.\end{equation*}

**ODE simulation**. To perform our simulations, we need to specify the initial values of the variables in the system, the parameter values, and the total simulation time. In this example, we will use the following values for the initial population levels:

**Table C3.** Initial Conditions.

Variable	Initial Conditions	Units
*R*_*n*_	0	Molecules
*R*_*c*_	0	Molecules
*P*	0	Molecules

and the following values for the model parameters:

**Table C4.** Parameter values.

Parameter	Starting value	Units
*k*_*r*_	2	min^−1^
*k*_*t*_	0.083	min^−1^
*k*_*p*_	0.5	min^−1^
*γ*_*r*_	0.05	min^−1^
*γ*_*p*_	0.25	min^−1^

**Figure C5.**
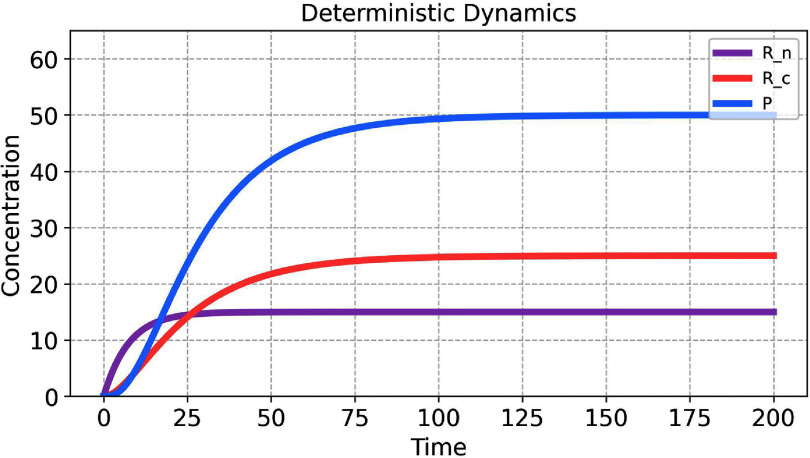
Deterministic simulation output for the biochemical system. Species *P*, *R_n_*, and *R_c_* rapidly achieve a steady-state concentration, indicating a saturating process or equilibrium is reached quickly. This deterministic approach provides a baseline understanding of the system’s dynamics, allowing for the identification of steady states and thresholds.

Figure C5 shows the solution of the system using the parameter values and initial conditions given in the tables above.

#### 5.4.1. Stochastic simulation approach

The continuous and deterministic approach discussed in section 5.3.1 fails to capture variations in system behavior due to the discrete nature of genes, RNA, and proteins or the randomness and biochemical noise that stems from the stochastic timing of bio-molecular events. Reproducing such phenomena requires a discrete description of the process and consideration of the distributions of event times. For this, we often employ *KMC* methods that use one or more random number generators to sample the time evolution as the process moves between different points in the discrete state space.

One such KMC approach is Gillespie’s *direct* ***stochastic simulation algorithm (SSA)*** [84–86]. The SSA simulates the process one reaction at a time, deciding at each step when the next reaction occurs and how the system’s state is affected by that reaction. The SSA is defined in terms of the same stoichiometry matrix, **S**, and propensity functions, **W**, introduced above. It seeks to simulate the system from some initial state, $\mathbf{x}(0)$, until some final time, $t_\textrm{max}$, through a series of jumps whose random times and directions are chosen from probability distributions defined by **S** and **W**.

Let $a_0(\mathbf{x}) = \sum_{\mu = 1}^{M} w_{i}(\textbf{x})$ denote the current sum of all propensity functions. Under this definition, the time of the next reaction has an exponential distribution ${\tau}\sim \mathrm{Exp}(a_0(\mathbf{x}))$, and a sample of this distribution is easily generated by sampling a uniform random number $u_1\sim U(0,1)$ via the transformation:

\begin{equation*} {\tau} = \frac{1}{{a}_0\left(\textbf{x}\right)} \log\frac{1}{u_1}.\end{equation*}

Furthermore, the index of the chosen reaction has a categorical distribution of $k \sim \mathrm{Cat}\{w_1/a_0,\ldots,w_{M}/a_0\}$ that can be found by sampling a second uniform random variable, $u_2 \sim U(0,1)$, and finding the smallest *k*, such that:

\begin{equation*} \sum_{\mu = 1}^{k} w_{\mu}\left(\textbf{x}\right)\unicode{x2A7E} u_2 {a}_0\left(\textbf{x}\right).\end{equation*}

Once samples of *τ* and *k* are generated, one can update to the new current time ($t = t+{\tau}$), and if this time is less than $t_\textrm{max}$, the state of the system can be updated to ($\textbf{x} = \textbf{x}+\textbf{s}_k$). This process continues until *t* exceeds $t_\textrm{max}$. To compute many trajectories, the process is repeated with new sequences of random numbers. Although easy to implement, the SSA can be computationally intensive, thus motivating the creation of more computationally efficient methods that approximate the SSA and generate stochastic trajectories, including the *τ-Leaping* Algorithm [87] and the *Chemical Langevin equation* Algorithm [83]. These methods, however, may result in a large loss of accuracy if the system species have low numbers of molecules or if the propensity functions experience quick, substantial changes.

The interested reader is invited to review the Python Tutorial and lecture videos on the SSA via the links in the appendix and to attempt to build an SSA model in the following example.

### 5.5. UQ-Bio Summer school challenge—stochastic simulation of gene regulation

The next step is to extend the model from before to implement the Gillespie SSA to model the stochastic dynamics of the system. Assign initial molecule counts and reaction rate constants and simulate the process for 100 repetitions to capture the variability inherent in the system. Generate histograms and time-course plots to illustrate the distribution and variability of the species. Compare the stochastic simulation results with deterministic ODE results to explore the impact of noise on the system dynamics.

Finally, extend the model to consider a drug perturbation inhibits mRNA transport at a prescribed time and amount. Formulate your model in terms of a parameter $\alpha\in[0,1]$ that captures the efficacy of the drug, where *α* = 0 denotes no effect (i.e. RNA transport occurs at the unperturbed rate) and *α* = 1 denotes perfect effect (i.e. RNA transport is fully repressed). After extending the mode, simulate the temporal effects of the drug in your system using both deterministic and stochastic dynamics, and show how the system adapts from the no-drug steady state to the new steady state under a 90% effective drug treatment.

#### Solution—stochastic simulations

**Figure C6.**
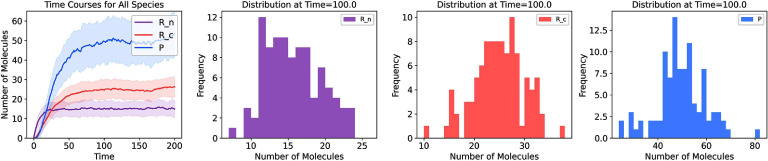
Stochastic simulation analysis. A set of 100 individual trajectories were run. Each trajectory was used to build the median and standard deviation plotted on the time course in the top-right corner figure. A distribution of the values is given for each variable in the system. The simulation was performed using Gillespy2, [88]. Initial conditions, parameter values, and time span are reported in the tables above. Histograms were built by taking the species concentrations at time 100 of the simulation.

To solve the system under stochastic dynamics, we implement the SSA [85]. For this, we can implement the SSA by ourselves or use a library such as Gillespy2 [88]. In this step, ensure that your model is in the correct format that the library or your code requires. Figure C6 shows the temporal dynamics for all the variables in the system. In contrast to deterministic dynamics, the stochastic simulation returns different values for each run. It is common to run multiple repetitions (trajectories) to quantify the system variability. The simulation was performed for 100 independent trajectories, and the statistics of the different species can be obtained from the histograms representing the amount of particles for each species.

#### Solution—simulating drug perturbations

To model the effect of a drug that inhibits the mRNA transport, we employ a mathematical approach that allows the parameter *k*_*t*_, representing the rate of mRNA transport, to vary in response to drug application. This approach simulates conditions where the drug, once introduced, exerts a constant inhibitory effect throughout the duration of the simulation. The parameter *k*_*t*_ is re-defined as follows:

\begin{equation*} k_{t}\left(t\right) = \begin{cases} k_{t} &amp; \textrm{if } t < t_\mathrm{drug} \\ \alpha \cdot k_{t} &amp; \textrm{if } t \unicode{x2A7E} t_\mathrm{drug}. \end{cases}\end{equation*}

Here, *k*_*t*_ represents the baseline rate of mRNA transport in the absence of the drug. The parameter $\alpha\in [0,1]$ modulates the rate of mRNA transport in response to the drug’s presence. That is, a value of *α* = 0 would indicate complete inhibition of the mRNA transport process by the drug, while *α* = 1 would reflect no change in the transport rate. The time $t_\mathrm{drug}$ specifies the moment within the simulation timeline when the drug is introduced to the system.

**Figure C7.**
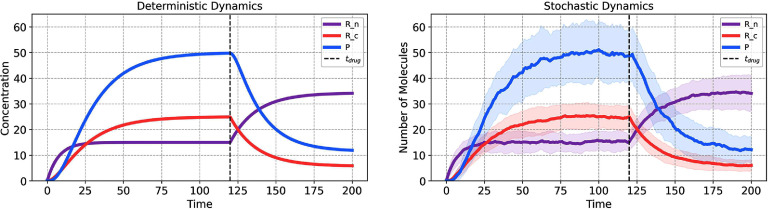
Effects of drug perturbation. Simulation of Drug inhibition at $t_\mathrm{drug} = 120$ min, $k_{t}(t < t_\mathrm{drug}) = 0.083$ min^−1^, and $k_{t}(t > = t_\mathrm{drug}) = 0.0083$ min^−1^. For stochastic simulations, 100 trajectories were run.

As a specific example, we reduced the transport rate as follows: $k_{t}(t > = t_\mathrm{drug}) = 0.1*k_{t}$. The application of the drug was simulated at time $t_\mathrm{drug} = 120$ for a total simulation time, $t \in [0, 200]$ min. The model was solved under deterministic and stochastic dynamics (figure C7, top left and right, respectively).

#### 5.5.1. Chemical master equation (CME) based approach

Using the SSA from the previous section, one can generate large sets of trajectories, where each trajectory is one statistical sample of the process behavior. However, because the process is random, every trajectory will be different, and none should be expected to match exactly to any real collection of data. To compare SSA models to actual data, one must generate large numbers of such samples at times and conditions of interest and then combine these data to form histograms. These histograms, when normalized to sum to one, approach the probability distributions of the system response at each point in time. For example, if one is interested in the probability of any particular state, **x**_*i*_, at some time, *t*, we can estimate the probability of that state:

\begin{align*} p\left(\mathbf{x_i},t\right) &amp; = \frac{\textrm{Number of trajectories at}\ \mathbf{x}_i\ \textrm{at time}\ t}{N}+ \mathcal{O}\left(N^{-1/2}\right),\end{align*}

where *N* is the number of SSA trajectories, and $\mathcal{O}(N^{-1/2})$ denotes the convergence rate of the error. Unfortunately, if there is a large number of possible states, then **x**_*i*_ may be rare and $p(\mathbf{x_i},t)$ could be very small. Because error in the estimate (equation (14)) converges with order $1/\sqrt{N}$, to obtain an accurate estimate for $p(\mathbf{x_i},t)$, one may need extremely large numbers of simulations.

Another approach to analyze the statistics of such processes is to define the Chemical Master equation (CME, also known as the *forward Kolmogorov equation*). The CME is a system of linear ODEs that directly describes the changes in the probability mass through time, $p(\mathbf{x_i},t)$, for all possible values for all **x**_*i*_ that can be reached by the system. When solved, the CME contains all the information to fully describe the evolution of the system’s statistics, forgoing the need to generate SSA samples. Using standard notation, the CME can be written in terms of reactions that result in transitions out of state **x**_*i*_ and reactions that result in a transition into state **x**_*i*_ as follows:

\begin{align*} \frac{\mathrm{d}}{\mathrm{d}t}p\left(\mathbf{x_i},t\right) &amp; = \underbrace{-\sum_{\mu = 1}^{M} p\left({\mathbf{x_i}},t\right) w_{\mu}\left(\mathbf{x_i},t\right)}_{\textrm{Transitions FROM state }\mathbf{x_i}}+ \underbrace{\sum_{\mu = 1}^{M} p\left({\mathbf{x_i}} - \mathbf{s}_{\mu},t\right) w_{\mu}\left({\mathbf{x_i}} - \mathbf{s}_{\mu},t\right)}_{\textrm{Transitions TO state }\mathbf{x_i}}.\end{align*}

Consistent with the notation described in section 5.1, the probability mass vector for the full state space can be defined as $\textbf{P}\equiv{[p({\mathbf{x_1}}), p({\mathbf{x_2})},\ldots]^\textrm{T}}$, and the CME can be written in a more condensed matrix form as:

\begin{equation*} \frac{\mathrm{d}}{\mathrm{d}t}\textbf{P}\left(t\right) = \textbf{AP}\left(t\right),\end{equation*}

where the *infinitesimal generator matrix*, **A**, is equal to:

\begin{equation*} \textbf{A}_{ij} = \left\{ \begin{array}{cl} -\sum_{\mu = 1}^{M}w_{\mu}\left(\mathbf{x_j}\right) &amp; \mbox{for}\ i = j,\\ w_{\mu}\left(\mathbf{x_j}\right) &amp; \mbox{for}~\left(i,j\right)~\mbox{such that}\ \mathbf{x_i} = \mathbf{x_j} + \nu_\mu,\\ 0 &amp; \mbox{otherwise} \end{array} \right\}.\end{equation*}

Unfortunately, it is important to consider that for most realistic systems, the state space is infinite, meaning that an infinite number of ODEs are needed to formulate the CME. For this reason, approximate solution schemes are needed to solve the CME.

#### 5.5.2. Finite state projection (FSP)

With the exception of very simple models, there is rarely an exact or analytical solution that can be computed, and the state space of the CME is often infinite or computationally intractable. To circumvent these issues, numerical approximations may be employed. One such technique is to project the CME state space onto a finite subset of states, naturally termed as *finite state projection* (FSP) [89–92]. FSP allows for manageable computation of the time evolution of the probability distribution over chemical reaction states while keeping track of, and enforcing bounds upon, the error that results from the truncation of the whole CME state space [93]. Additionally, the error threshold can be adjusted, and the size of the FSP subset iteratively contracted and expanded to find the optimal projection with high accuracy and low cost on computational efficiency.

Mathematically, the FSP method can be defined as follows: Let $\mathbf{X} = \{\mathbf{x_1}, \mathbf{x_2}, \ldots\}$ denote any enumeration of all possible states that can be reached by the system. Next, define $\textbf{J} = \{j_1,j_2,\ldots,j_N\}$ as a finite set of indices, so that the set $\mathbf{X_J} = \{\mathbf{x_{j1}},\ldots,\mathbf{x_{jN}}\}$ is a finite set of states. Also, let the complement set $\mathbf{X}_\mathbf{J^{^{\prime}}}$ contain all of the states that were *not* included in $\mathbf{X_J}$. Using this notation, the full CME can be reordered as:

\begin{equation*} \frac{\mathrm{d}}{\mathrm{d}t} \left[ \begin{array}{cl} \textbf{P}_J\left(t\right) \\ \textbf{P}_{J^{^{\prime}}}\left(t\right) \\ \end{array} \right] = \left[ \begin{array}{cl} \textbf{A}_{JJ} &amp; \textbf{A}_{JJ^{^{\prime}}} \\ \textbf{A}_{J^{^{\prime}}J} &amp; \textbf{A}_{J^{^{\prime}}J^{^{\prime}}} \\ \end{array} \right] \left[ \begin{array}{cl} \textbf{P}_J\left(t\right) \\ \textbf{P}_{J^{^{\prime}}}\left(t\right) \\ \end{array} \right].\end{equation*}

In the FSP approach, the second set of states is replaced by an absorbing sink, resulting in a new *finite* dimensional master equation:

\begin{equation*} \frac{\mathrm{d}}{\mathrm{d}t} \left[ \begin{array}{cl} \textbf{P}^{\textrm{FSP}}_J\left(t\right) \\ g\left(t\right) \\ \end{array} \right] = \left[ \begin{array}{cl} \mathbf{A_{JJ}} &amp; \textbf{0} \\ \mathbf{-1^TA_{JJ}} &amp; 0 \\ \end{array} \right] \left[ \begin{array}{cl} \textbf{P}^{\textrm{FSP}}_J\left(t\right) \\ g\left(t\right) \\ \end{array} \right]\end{equation*}

where *g*(*t*) records the amount of probability mass to reach the absorbing sink. In this formulation of the master equation, once probability mass leaves $\mathbf{X_J}$, it cannot return and remains in the absorbing state, and *g*(*t*) therefore provides the *exact probability mass* that has left $\mathbf{X_J}$ as a function of time.

In addition to providing an exact calculation for escape times, the FSP solution relates to the original CME solution in three important ways:

• First, the FSP is a lower bound on the exact CME solution ($\mathbf{P_J}^{\textrm{FSP}} \unicode{x2A7D} \mathbf{P_J}$) for every possible state of the process. (Note: for finite CME state space, the FSP provides an exact analytical solution.)
• Second, the exact total absolute error of the approximation is known, $\left|\mathbf{P_J} - \mathbf{P_J}^{\textrm{FSP}}\right|_1 + \left|\mathbf{P_J}^{^{\prime}}\right|_1 = g(t)$.
• Third, the error of the FSP, *g*(*t*), decreases monotonically as one adds additional states to the set **J**.

With these three guarantees, one can then specify a maximum tolerable CME error as a monotonically increasing function, $\varepsilon(t) > 0$, where $\varepsilon(t_1)\unicode{x2A7D}\varepsilon(t_2)$ for every $t_2 > t_1$, and implement the FSP algorithm as follows [89, 92, 93]:

• An initial state space **X**_J_ is selected, and the initial distribution along this space is extracted from $\textbf{P}(0)$.
• The subsequent probabilities, $\textbf{P}(t)$, and the FSP approximation error, *g*(*t*), are integrated forward in time using equation (19). If *g*(*t*) exceeds a user-defined threshold function $\epsilon(t)$ at any time, the integration is paused, and more states are added to **X**_J_ before continuing the integration.
• The process is repeated until the final time, *t_f_*, at which point the approximation guarantees that the FSP solution is below the error threshold at all times.

The interested reader is invited to review the tutorial videos and Python Notebook provided in the appendix and the following example for further practice using the CME and FSP approaches to solve for gene regulation probability distributions.

### 5.6. UQ-Bio Summer School challenge—finite state projection analysis of gene regulation

The next step is to solve the gene regulation model system (section 5.2) using the FSP to approximate the CME solution. To this end, assume the same initial molecule counts and reaction rate constants as before, assume that the same inhibition process was simulated in drug application at time $t_\mathrm{drug} = 120$ min, and simulate the system over the time interval $t\in[0,200]$ min. The FSP solution provides the full probability distribution for each species at each time point. From these distributions, plot the mean and standard deviation of the molecular counts versus time, as well as the joint probability distributions for nuclear mRNA, cytoplasmic mRNA, and protein at the final time. Finally, compare your results with the deterministic and SSA solutions.

#### Solution—simulating the FSP

To model the system using the FSP approach, we implemented the FSP using the algorithm provided in the Github repository given in the appendix. The FSP projection space used in the simple Python implementation (which does not automatically adjust the size of the subset based on error) was limited to the set ($R_n\unicode{x2A7D} 80$, $R_c\unicode{x2A7D}80$, $P\unicode{x2A7D} 120$ array, resulting in an infinitesimal generator matrix **A** with 793 881 rows and columns. The initial probability mass vector, **P**_0_ was set as one for the given initial state ($R_n = 0$, $R_c = 0$, *P* = 0) and zero for all other states. Equation (19) is then solved using a Python implementation of Expokit [94] that uses a Krylov subspace approximation for exponentiation of large sparse matrices. We note that the codes provided in Python are meant primarily for educational purposes and are not intended for computationally intensive investigations. For example, this FSP calculation in Python took a computation time of 1 m 20.3 s on Apple M3 Pro, while a more established implementation in MATLAB (known as the Stochastic System Identification Toolkit and available at https://github.com/MunskyGroup/SSIT/) runs in approximately 0.032 s on the same computer and with the same bounds.

Upon solving the FSP for the joint distribution of all species, $\mathbf{Pr}(R_n,R_c,P)$, it is straightforward to calculate model expected values (e.g. means) according to:

\begin{align*} \mu_n = \mathbb{E}\left\{R_n\right\} &amp; = \sum_{i,j,k} i \mathbf{Pr}\left(R_n = i,R_c = j,P = k\right),\nonumber\\ \mu_c = \mathbb{E}\left\{R_c\right\} &amp; = \sum_{i,j,k} j \mathbf{Pr}\left(R_n = i,R_c = j,P = k\right),\nonumber\\ \mu_p = \mathbb{E}\left\{P\right\} &amp; = \sum_{i,j,k} k \mathbf{Pr}\left(R_n = i,R_c = j,P = k\right).\end{align*}

Similarly, the uncentered moments of $\mathbb{E}\{R_n^a R_c^b P^c\}$ are calculated by:

\begin{equation*} \mathbb{E}\left\{R_n^a R_c^b P^c\right\} = \sum_{i,j,k} i^a j^b k^c \mathbf{Pr}\left(R_n = i,R_c = j,P = k\right),\end{equation*}

and variances and covariances are easily calculated as:

\begin{equation*} \sigma_{xy} = \mathbb{E}\left\{xy\right\} - \mathbb{E}\left\{x\right\}\mathbb{E}\left\{y\right\}\end{equation*}

where *x* and *y* can be replaced with different combinations of *R_n_*, *R_c_* and *P*.

**Figure C8.**
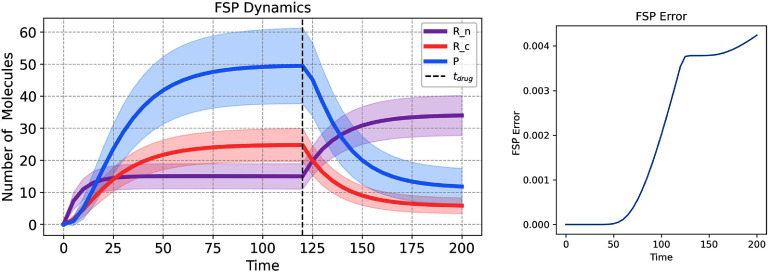
FSP Solution and Error. (Left) Simulation of drug inhibition at $t_\mathrm{drug} = 120$ min, with $k_{t}(t < t_\mathrm{drug}) =$ $0.083$ min^−1^ and $k_{t}(t\unicode{x2A7E} t_\mathrm{drug}) = 0.0083$ min^−1^. (Right) FSP truncation error vs. time calculated as final term, *g*(*t*) in equation (19).

Using these calculations, figure C8 (left) shows the time course of the mean and standard deviation for each of the three species, which are in excellent agreement with the results from sections 5.4 and 5.5. Figure C8 (right) shows the FSP error versus time for the chosen model and choice of projection space, demonstrating that the total error is about 0.004 at the final time. To find the 2-species joint distributions, we simply marginalize over the left out species according to:

\begin{align*} \mathbf{Pr}\left(R_n = i,R_c = j\right) &amp; = \sum_k \mathbf{Pr}\left(R_n = i,R_c = j,P = k\right),\nonumber\\ \mathbf{Pr}\left(R_n = i,P = k\right) &amp; = \sum_j \mathbf{Pr}\left(R_n = i,R_c = j,P = k\right),\nonumber\\ \mathbf{Pr}\left(R_c = j,P = k\right) &amp; = \sum_i \mathbf{Pr}\left(R_n = i,R_c = j,P = k\right).\end{align*}

**Figure C9.**
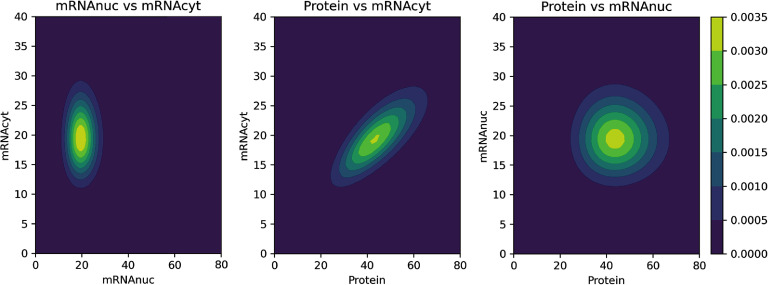
The 2-species joint probability contours shown for each combination of the model species (*R_n_*, *R_c_*, *P*) at $t_\mathrm{drug}$ = 120 min.

Figure C9 shows the joint probabilities between each pair of the three species calculated at the time *t* = 120 (min).

## 6. Inferring model parameters from experimental data

With quantitative single-cell data (sections 3 and 4) and a solvable computational model (section 5), the next step is to use those data to constrain parameters of the model. In this section, we discuss a few of the simplest tools to fit models to data and extract parameters.

***Model and parameter identification*** is the process of finding mechanisms or parameter sets that best describe or predict observed data. It is akin to finding the best-fitting line or curve that represents the relationship between variables in the dataset. Central to this process is the ***likelihood function***, which quantifies the probability of observing the data under the specific set of model parameters [95]. Intuitively, we want to find those parameters that make the observations as plausible as possible, those that maximize the likelihood.

For example, consider observed data $\mathcal{D} = (d_1, d_2, \ldots, d_n)$ for a specific cell measured at time points $\mathbf{t} = [t_1, t_2, \ldots, t_n]$. The likelihood function is

\begin{equation*} L\left(\mathcal{D}|\boldsymbol{\theta}\right) = \textrm{Pr}\left(d_1, d_2, \ldots, d_n \, | \, \boldsymbol{\theta}\right).\end{equation*}

Note that here the data $\mathcal{D}$ is fixed, and the likelihood depends on the parameters ***θ*** only.

For problems where an exact CME solution is computationally tractable, one can estimate the log-likelihood function in equation (24) directly. More generally, whenever the CME can be solved exactly or approximately using the FSP, likelihoods such as that in equation (24) can be computed. The most common way to do so is to rewrite the equation as a sequence of conditional probabilities:

\begin{align*} L\left(\mathcal{D}|\boldsymbol{\theta}\right)&amp;=\textrm{Pr}\left(d_1|\boldsymbol{\theta}\right) \cdot \textrm{Pr}\left(d_2|d_1,\boldsymbol{\theta}\right)\, \cdots\, \textrm{Pr}\left(d_n|d_1,d_2,\ldots,d_{n-1},\boldsymbol{\theta}\right).\end{align*}

Solving the CME up to time *t*_1_ allows us to compute $\textrm{Pr}(d_1|\boldsymbol{\theta})$, the probability distribution at the first point. Then, conditioning on the first observation and continuing the solution up to time *t*_2_ allows us to compute the second term, $\textrm{Pr}(d_2|d_1,\boldsymbol{\theta})$. This can be iterated for all time steps to arrive at the total likelihood (equation (25)). The details of this algorithm (which is generically called the forward algorithm for state-space models) can be found in [96, 97]. The general procedure of computing likelihoods of time-series as in equation (25) is called filtering, which includes the famous Kalman filter [98] and the HMM filter, another name for the algorithm outlined above.

Observing a single cell does not yield much information about a system, especially for highly stochastic processes where cells can exhibit very variable behavior. For this reason, one often observes multiple independent cells at the same time; as long as these cells do not affect each other during the experiment, one can obtain the likelihood for all cells by multiplying the likelihoods in equation (24) for each cell. As a result, the computational effort for computing the likelihood increases linearly with the number of cells, but this is often a worthy trade-off given the improved estimates one usually gets. For many experimental approaches, such as smFISH or sequencing, one can only obtain a single snapshot measurement for each cell. In this case, if one assumes that every cell measurement is independent, where *d_i_* refers to the $i\textrm{th}$ cell, then the conditional distributions in equation (25) reduce to:

\begin{align*} L\left(\mathcal{D}|\boldsymbol{\theta}\right) &amp; = \textrm{Pr}\left(d_1|\boldsymbol{\theta}\right) \cdot \textrm{Pr}\left(d_2|\boldsymbol{\theta}\right) \, \cdots \, \textrm{Pr}\left(d_n|\boldsymbol{\theta}\right) = \prod_{i}\textrm{Pr}\left(d_i|\boldsymbol{\theta}\right).\end{align*}

Unfortunately, for reaction networks with many species or large particle numbers, even approximately solving the CME using the FSP may be infeasible, and other approaches have to be used. One common scenario is where all species have large populations—in this case, the distribution of particle numbers approaches a Normal distribution following a version of the Central Limit Theorem [99]. This is called the linear noise approximation (LNA). In this regime, the probability of the observed data can be approximated by a product of Gaussian distributions:

\begin{align*} &amp; \mathrm{Pr}\left(\boldsymbol d |\boldsymbol{\theta}\right) = \frac{1}{\sqrt{2\pi |\Sigma|^2}} \exp\left(-\frac 1 2 \left(\boldsymbol d - \boldsymbol \mu\right)^T \Sigma^{-1} \left(\boldsymbol d - \boldsymbol \mu\right)\right).\end{align*}

Here, $\mu_j = y(t_j,\boldsymbol{\theta})$ is the deterministic model prediction at time *t_j_* as computed using section 5.3.1, which is the mean used in the Linear Noise Approximation, and Σ is the covariance matrix across different time points. The terms in equation (27) can all be computed by solving systems of ODEs (see [100]). Standard approaches to compute the likelihood use an analog of the forward algorithm described above, replacing the CME with the LNA between observations. In cases where a suitable approximation like the LNA is not available, parameter estimation becomes more difficult. Simulation-based inference methods, such as Approximate Bayesian Computation [101], can be used in this situation, although they are often computationally very expensive.

No matter how the likelihood is computed, the next step in inference is typically finding parameters that best fit the data, as measured by the likelihood. The most straightforward way to do so is to compute the maximum likelihood estimate (MLE):

\begin{equation*} \hat{\boldsymbol{\theta}} = \underset{\boldsymbol{\theta}}{\arg \max} \, L\left(\mathcal{D}|\boldsymbol{\theta}\right),\end{equation*}

where ${\textrm{argmax}}$ represents the argument that returns the maximum value of the function. In practice, this can be found by using standard optimization routines, such as those available in scipy.optimize in Python. It is important to note that this optimization problem is not straightforward; the likelihood function is often hard to compute, prone to local maxima, and often does not admit a single global maximum. This is often the case if the model has too many parameters for the amount and type of data collected, a phenomenon called ***parameter unidentifiability*** [102].

On a separate note, the maximum likelihood parameters are often not the ‘true’ parameters of the underlying model—this is because the systems we study are stochastic, so a certain amount of noise will affect our experimental outcomes. This phenomenon is called parameter uncertainty, and we will turn to this problem next.

### 6.1. Parameter uncertainty

From a technical perspective, it is important to keep in mind that biological experiments are often difficult to perform, and due to the delicate nature of living cells, we cannot directly measure many of the things we care about. When we do manage to measure them, these measurements are often subject to a lot of noise, both noise intrinsic to the system we want to model as well as technical (measurement) noise. Therefore, we must be careful about drawing precise conclusions given imperfect data. In the context of inference, this problem appears when one conducts maximum likelihood estimation several times for identical systems; each run will suggest different parameters simply because measurements will differ between experiments. To get an idea of how reliable parameter estimates are, therefore, one needs to consider the problem of parameter uncertainty.

There are various statistical ways of dealing with parameter uncertainty, most commonly divided between frequentist, likelihood, and Bayesian approaches.

In ***frequentist statistics***, probability is accumulated over repetitions of trials or experiments, counting the frequency of observations over the long term. For example, the frequency of a flipped coin landing on ‘heads’ over a large number of trials is directly interpreted as its probability, and likewise for the number of SSA trajectories visiting particular states in the CME.

***Likelihood-based statistics*** diverge from the traditional frequentist perspective in that we assume that there is a true underlying parameter set that gave rise to our observed data, and the likelihood of observing said data is computed as a function of various parameter sets. In other words, the likelihood function serves to measure the amount of support the data shows for different combinations of parameter values. MLEs [95] point to the set of parameters that maximizes the likelihood function $L(\boldsymbol{\theta};\mathcal{D})$, that is, the highest conditional probability $P(\mathcal{D}|\boldsymbol{\theta})$.

In the likelihood approach, measurements of uncertainty surrounding our likelihood estimates are then computed subsequently. Examples of methods to quantify our uncertainty about our answer include: the construction of confidence intervals that are meant to capture the estimated parameters across multiple independent data sets and their MLEs *λ*% of the time (e.g. 95%) [103]; using likelihood ratios to compare models and thereby gain some insight into the model uncertainties [104]; bootstrap analyses that resample either real or simulated data (non-parametric and parametric bootstrapping, respectively), recomputing the MLEs for each resample [105]; and computing the Cramér-Rao bound [106, 107] and Fisher information matrix to elucidate lower bounds on variance, covariances between different parameter estimates, and asymptotic variances of the individual components of the MLE variance [95, 108].

***Bayesian statistics***, on the other hand, incorporates any prior information we may have obtained from previous analyses with regard to the model parameters in the form of prior probabilities $P(\boldsymbol{\theta})$. This updates our beliefs and our degree of uncertainty about the updated parameter values inferred from our new set of experimental data $\mathcal D$ in the form of a posterior probability distribution $P(\boldsymbol{\theta} | \mathcal{D})$. This updating step is again based on the likelihood function $L(\boldsymbol{\theta};\mathcal{D})$, expressed as the conditional probability $P(\mathcal{D}|\boldsymbol{\theta})$, from equation (25). Intuitively, the probability we assign to parameters ***θ*** is weighted by how plausible they make the data appear.

The precise formula is called ***Bayes’ formula***:

\begin{equation*} P\left(\boldsymbol{\theta} | \mathcal{D}\right) = \frac{P\left(\mathcal{D}|\boldsymbol{\theta}\right) \cdot P\left(\boldsymbol{\theta}\right)}{P\left(\mathcal{D}\right)},\end{equation*}

with a marginalization term $P(\mathcal D)$ that can be referred to variously, for example, as the marginal likelihood, model evidence, or prior predictive distribution. $P(\mathcal{D})$ represents all possible occurrences of the data $\mathcal{D}$ and can be tricky to obtain, so it is often disregarded to yield the proportional posterior probability distribution:

\begin{equation*} P\left(\boldsymbol{\theta} | \mathcal{D}\right) \propto P\left(\mathcal{D}|\boldsymbol{\theta}\right) \cdot P\left(\boldsymbol{\theta}\right).\end{equation*}

Bayesian measures of uncertainty come ‘for free’ in the inference of the posterior distribution, whose properties, such as variance and deviation, can be examined for such insights, whether exact or proportional, and additional measures may be included *a posteriori*. For example, credible intervals are the Bayesian equivalent of confidence intervals and can be used to showcase how often a Bayesian model captures a true parameter value. Bayes Factors are roughly equivalent to the likelihood ratio, and can be used to compare models and characterize model uncertainty [109, 110].

While being a principled and flexible way to treat statistical uncertainty, Bayesian inference suffers from comparatively more computational difficulties than other approaches. Most notably, the need to consider not only one set of parameters, but rather an entire probability distribution over such parameter sets, renders it more complex. In particular, computing equation (30) is difficult since the proportionality factor is not usually known—this is given by the requirement that the left-hand side, being a probability distribution, must integrate to one (i.e. over all parameters ***θ*** in the set **Θ**). One therefore needs to compute:

\begin{equation*} P\left(\mathcal{D}\right) = \int_{\boldsymbol{\Theta}} P\left(\mathcal{D}|\boldsymbol{\theta}\right) \cdot P\left(\boldsymbol{\theta}\right) \mathrm{d} \boldsymbol{\theta},\end{equation*}

which quickly becomes intractable for complex models. Nevertheless, there is an abundance of algorithms that allow us to perform Bayesian inference without having to compute equation (31) directly, the most important of which we will introduce in the next section. Historically neglected due to its unfavorable computational demands, Bayesian inference has become an increasingly widespread methodology for quantitative modeling in the sciences with the development of modern computing power. We refer to the excellent textbooks (McElreath, Statistical Rethinking [111]) and (Gelman, Bayesian Data Analysis [112]) for a more detailed exposition of Bayesian ideas.

### 6.2. The Metropolis–Hastings algorithm

A numerical method commonly used to estimate the unnormalized posterior distribution, $P(\boldsymbol{\theta} | \mathcal{D})$, is the Metropolis–Hastings (MH) algorithm. This algorithm is an example of Markov Chain Monte Carlo (MCMC), currently the most widespread method to approximate an arbitrary target distribution, such as a posterior in Bayesian inference [113]. Here, a Monte Carlo method is any algorithm that uses random variables to estimate a quantity of interest (such as a probability distribution), and Markov Chains provide the means of generating these random variables.

A ***Markov chain*** is any sequence of random variables, $X_0, X_1, X_2, \ldots,$ such that at each point, the next value $X_{n+1}$ only depends on the current value *X_n_*; that is, a random walk with no memory. A Markov chain is completely defined by the initial distribution over *X*_0_, along with the transition distributions $q(X_{n+1}|X_n)$.

For any given Markov chain, one can compute the distributions $p(X_0),\,p(X_1),\,p(X_2),\ldots$ for all times, starting with the initial distribution. In many cases, one observes that these distributions start to become more and more similar to each other as *n* increases, approaching an asymptotic distribution which we will call $\pi(X)$. This distribution, when it exists, is called the stationary distribution of the Markov chain. MCMC works by constructing a Markov chain with simple transition distributions $q(X_{n+1}|X_n)$ that has the desired target distribution as its stationary distribution. Once this is given, we can draw (approximate) samples from the target distribution by simulating this Markov chain for long enough.

The trick with MCMC is to find a Markov chain that is easy to simulate yet converges to the desired target distribution, which can be arbitrarily complex. Different algorithms vary in how this Markov chain is constructed, but the MH algorithm underlies a large portion of these methods. The biggest advantage of MH is that it does not require the normalization constant (31), which makes it directly applicable to many Bayesian inference problems. Deriving the MH algorithm is outside the scope of this paper, but good references can be found in [113, 114] (Mackay, Information Theory, Inference, and Learning Algorithms, Chapter IV).

However the parameter uncertainty $P(\boldsymbol{\theta})$ is quantified, it can then be used with the model to obtain several important insights into the expected qualities of the identified model and its predictions. For example, highly uncertain combinations of parameters (sometimes called sloppy parameters [115]) can call attention to aspects of the model that are poorly constrained by the existing data. Alternatively, when making predictions for responses in new circumstances, one can sample over the parameter uncertainty to generate a range of potential results, thus quantifying how the uncertainty propagates from the model into its predictions. For some circumstances, the predictions may be very tight despite large amounts of parameter uncertainty and, for others, the predictions could be highly variable, thus providing insight into the range of experimental circumstances over which the model can be trusted (i.e. where predictions are tight) or identifying new experiments that could provide better information to constrain parameters (i.e. where predictions are variable).

The interested reader is invited to review the video tutorial and Python notebooks on Bayesian Thinking and Markov Chain Monte Carlo provided in the appendix and to attempt the final stage of the UQ-Bio Summer School challenge in the following section.

### 6.3. UQ-Bio Summer School challenge—infer model parameters and their uncertainties

Use the simulated experimental data provided at https://github .com/luisub/qbio_paper.git to estimate the parameters of the model. First search over parameter space to find the MLE, and then assume a prior for reasonable values for each parameter in your model, and implement the MH algorithm to sample sets of parameters that are consistent with the experimental data. Make scatter plots of these samples to visualize the uncertainty of the model parameters.

#### Solution: quantifying model uncertainties

Using the provided simulated dataset, parameter values were estimated using the MH algorithm. For our analysis, we let $O_m^{\textrm{data}}(t_i)$ denote the simulated experimental data for the $m\textrm{th}$ species and the $i\textrm{th}$ time point, and we let $O_m^{\textrm{model}}(t_i)$ denote results from the ODE analysis developed in section 5.4 and evaluated at the same time points. The prior distribution of parameters was built assuming a lognormal distribution with a location *µ* of -1 and a scale *σ* of 2 for each parameter (i.e. all parameters were guessed to be independent and most likely to be within about two orders of magnitude from the base value of 0.1). To achieve better sampling efficiency, all parameters were defined in $log10$ space.

The likelihood function for the problem is estimated (using the ODE model) as:

\begin{equation*} L\left(\boldsymbol{\theta}\right) = \left(\prod_{m = 1}^M \prod_{i = 1}^N \frac{1}{\sqrt{2\pi\sigma^{\textrm{data}}_{m}\left(t_i\right)^2}}\right) \exp\left(-\sum_{m = 1}^M \sum_{i = 1}^N \frac{\left(O^{\textrm{data}}_{m}\left(t_i\right) - O^{\textrm{model}}_{m}\left(t_i; \boldsymbol{\theta}\right)\right)^2}{2\sigma^{\textrm{data}}_{m}\left(t_i\right)^2} \right)\end{equation*}

where *N* is the total number of observations, *i* represents the measured time points, *M* is the number of observed molecular species, $\sigma_{m}^{\textrm{data}}$ represents the observations error for the $m\textrm{th}$ species in the experimental data, under an assumption that this observation error is independent at each time and for each species, and ***θ*** is the vector of model parameters. To simplify calculation and to improve numerical stability during the optimization process, the log-likelihood is defined as follows:

\begin{equation*} \log L\left(\boldsymbol{\theta}\right) = -\frac{1}{2} \sum_{m = 1}^M \sum_{i = 1}^N \log\left(2\pi\sigma^{\textrm{data}}_{m}\left(t_i\right)^2\right) - \sum_{m = 1}^M \sum_{i = 1}^N \frac{\left(O^{\textrm{data}}_{m}\left(t_i\right) - O^{\textrm{model}}_{m}\left(t_i; \boldsymbol{\theta}\right)\right)^2}{2\sigma^{\textrm{data}}_{m}\left(t_i\right)^2}.\end{equation*}

To simplify the optimization process, we focus only on the elements of the log-likelihood function that actually depend on the parameters ***θ***. This simplification results in a relatively simple sum-of-squares function that can now be used to adjust ***θ*** to fit the model to the observed data:

\begin{equation*} \log L\left(\boldsymbol{\theta}\right) \propto -\sum_{m = 1}^M \sum_{i = 1}^N \frac{\left(O^{\textrm{data}}_{m}\left(t_i\right) - O^{\textrm{model}}_{m}\left(t_i; \boldsymbol{\theta}\right)\right)^2}{2\sigma^{\textrm{data}}_{m}\left(t_{i}\right)^2}.\end{equation*}

**Figure C10.**
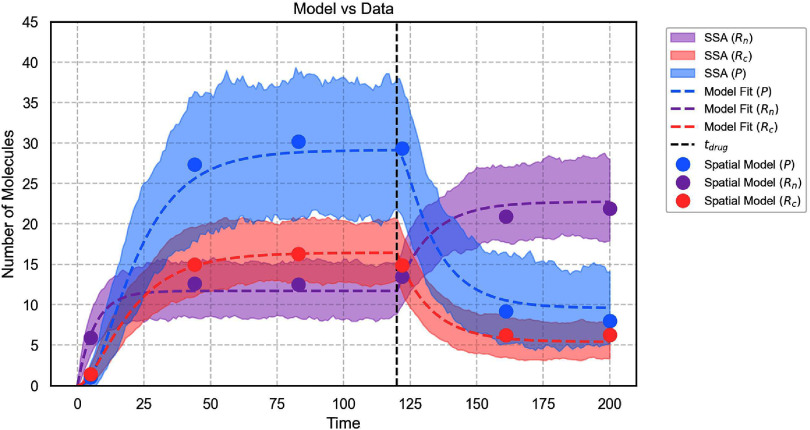
Model fit results. Comparison between deterministic ODEs and Stochastic Simulations using MLE parameters. Solid lines depict the deterministic ODE model predictions for the observable species: nuclear mRNA (*R*_*n*_), cytosolic mRNA (*R*_*c*_), and protein (*P*), based on the estimated parameter values. Data points (dots) represent the synthetic experimental observations for each species at six distinct time points. The shaded regions illustrate the variability across 200 stochastic simulation runs (SSA). Additionally, the vertical dashed line at (*t* = 120) time units indicates the time of drug application.

**Figure C11.**
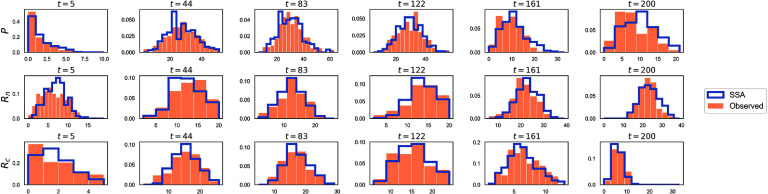
Model fit comparison between stochastic simulations and synthetic data. Solid blue lines SSA solution for the observable species: nuclear mRNA (*R*_*n*_), cytosolic mRNA (*R*_*c*_), and protein (*P*), based on the estimated parameter values. Data orange histograms represent the synthetic experimental observations for each species at six distinct time points. Histograms were built using 200 SSA runs.

We ran the deterministic model using the previously defined initial conditions and the mean values from the MH Chains to capture the system dynamics using the fitted parameter values. Figure C10 compares synthetic data and model results using the optimized parameter values, additionally, the SSA model was simulated using the obtained parameters and it was compared with the synthetic data obtaining similar distributions as shown in figure C11.

**Figure C12.**
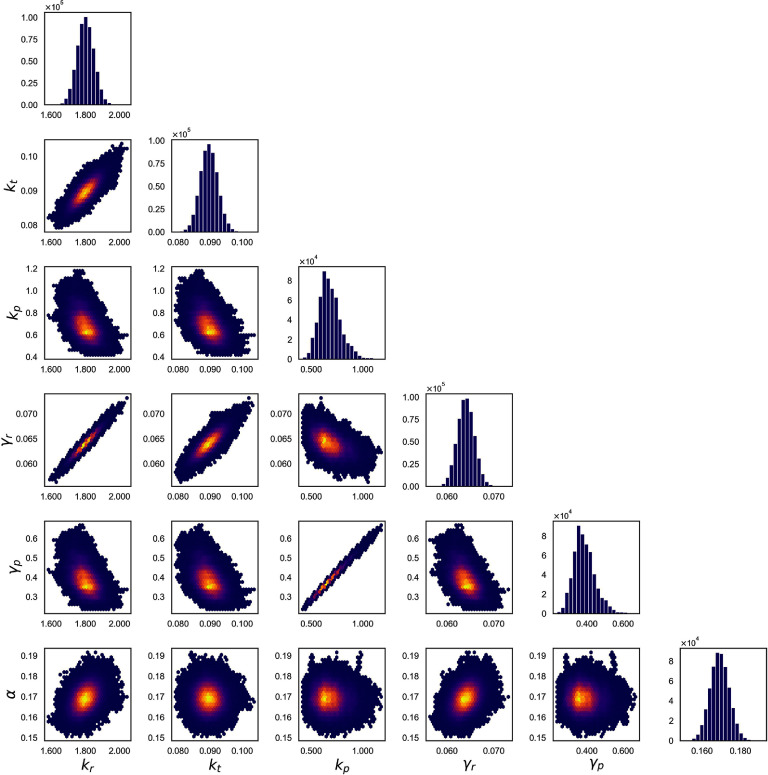
Parameter uncertainty. Histograms display the posterior distributions of the estimated parameters obtained from the Metropolis–Hastings Chains. The spread of each histogram reflects the uncertainty associated with each parameter estimate. Narrower distributions indicate higher confidence, while wider distributions suggest greater uncertainty.

Figure C12 shows distributions for the MH Chains for each estimated parameter. At the bottom, hexbin plots visualize the relationship between pairs of all estimated parameters. It can be observed that well-defined parameters reproduce a plot where values in the Metropolis–Hasting Chains are densely concentrated in the center of the plot. In contrast, undefined parameters reproduce elongated ellipses, indicating the estimated parameters can take any given values.

## 7. Conclusions and outlook

This review discussed the aims, history, and mathematical tools used in quantitative biology. By now, we expect the reader to understand the goals of mechanistic modeling and the adequate selection of the modeling approach based on the scientific hypothesis. We also expect the reader to understand the strengths and weaknesses of the different modeling techniques, but more importantly, the value of understanding that exact solutions are often impossible and approximated algorithms are available.

During the last two centuries, quantitative biology has been reinvented multiple times by adopting new experimental and computational technologies [32]. Nevertheless, during all these changes, it has been clear that combining mathematical models and experimental data has been central to breakthrough scientific discoveries. Modern biology uses high-throughput techniques, producing large data sets that need to be understood using formal quantitative approaches (for a comprehensive list of examples and citations, check the above section History of Quantitative Biology). The line dividing biology and computational biology is therefore blurring, and it is clear that all biology is in some way becoming computational biology [116].

Important advances have been made in understanding the central dogma during the last few decades, facilitated by the development of higher spatial and temporal resolution techniques. The low copy number of most molecules in the cell ensures the central importance of using single-cell and single-molecule resolution techniques, as well as the fundamental role of stochasticity in any molecular system [23]. In fact, stochasticity is one of the most important forces in determining gene expression, cell fate, and infection dynamics, to name a few [117]. Stochasticity is present in most biological systems, and mathematical methodologies have been developed to integrate it during the modeling approach [118]. This has led to the creation of powerful models used in the design of biological systems (i.e. the field of synthetic biology).

The aims of quantitative biology have gone beyond using mathematical models to reproduce experimental data and seek to understand the biological process in full detail [119]. Once a mathematical model is validated with experimental data, it becomes a powerful tool that can be used to integrate complex data sets, predict the system’s outcome in untested scenarios, and obtain the essential elements in a biological process [120]. It is thus clear that quantitative biology approaches achieve a greater understanding of the biological system beyond the sum of independent observations [121].

Quantitative biology has recently become heavily involved in experimental design, using modeling approaches to design more informative experiments. To this end, models are created with existing knowledge of the system, and the model predictions are used to design the next round of experiments. Then, experimental results are incorporated into the model to understand the systems better. In this iterative implementation, more system knowledge is gained in each iteration round. In particular, scientists are interested in determining the number of necessary experimental repetitions, the most informative time points to take measurements, and the best perturbations to consider. All of these design considerations pave a new way to perform experiments involving more quantitative planning [108, 122].

### 7.1. UQ-Bio Summer School challenge—summary and next steps

We have developed and analyzed a mathematical model to predict the impact of a drug that inhibits mRNA transport on gene expression at the single-cell level. We started by defining the model scope in section 2.3, a crucial preliminary step that helps to clarify the project goals before collecting or analyzing experimental data. We then processed simulated microscopy data in section 4.4 to extract quantitative information. In sections 5.2–5.6 we defined and simulated both deterministic and stochastic versions of the model, incorporating the drug’s effects. In 6.3, we used the experimental data to infer model parameters (using an assumption of the ODE model and Normally distributed errors) and we assessed the model’s fit. Using these analyses, one is now able to compare different models to one an other in the context of the data and the project goals and evaluate if the one or more models are sufficient to capture the biological system behavior. The next steps are open to the reader; these could involve (1) refining the model to include additional biological complexities (if the current model is insufficient), (2) expanding the inference approach to use the FSP approach to obtain a slower, but more accurate calculations of the likelihood function (3) reducing the complexity of the model to reduce uncertainty (if the data is limited or the model is too complex), (4) using the model to predict behaviors in new circumstances for which the model has high confidence (followed by experimental validation studies), or (5) using the model to design experimental conditions where the model predictions are less confident, or other modeling, prediction, or design tasks. For example, in the 2023 and 2024 UQ-Bio Summer School, teams of students considered several different drugs, identified mechanisms and parameters for each drug, and then were challenged to use their models to design a specific drug dosage regimen for the timing and concentration of each drug that would minimize protein expression while maintaining cell vitality. Then, on the final day of class, teams submitted their dosage regimens and live simulations were conducted to determine which team achieved the greatest level of success.

## Data Availability

The data that support the findings of this study are openly available at the following URL/DOI: https://github.com/luisub/qbio_paper.

## Appendix Links for online UQ-Bio Summer School resources

To aid with the mastery of the concepts discussed in this tutorial, we have created a set of tutorial notebooks in Python. The full set of notebooks and example data can be downloaded at https://github.com/MunskyGroup/uqbio2024 or https://doi.org/10.5281/zenodo.15442176. The repository consists of five modules including (1) Preliminary Python practice, (2) Image Processing, (3) Statistics, (4) Modeling Biochemical Processes, and (5) Bayesian Thinking and MCMC, although only Modules 2, 4, and 5 are discussed in this article. For those new to Python and to GitHub, the above website also includes instructions for installing Anaconda and setting up a Python environment, installing and getting started in VS Code, and working with GitHub.

### List of python tutorials

• **Tutorial on Basic Image Processing**—https://github.com/MunskyGroup/uqbio2024/blob/main/Module2-ImageProcessing/M2A_Basic_Image_Processing.ipynb. This tutorial covers introductory material for loading and manipulating images from section 4.
• **Tutorial on Segmenting Images**—https://github.com/MunskyGroup/uqbio2024/blob/main/Module2-ImageProcessing/M2B_Segmenting_Images.ipynb. This tutorial covers cell segmentation methods discussed in section 4.
• **Tutorial on Tracking Particles**—https://github.com/MunskyGroup/uqbio2024/blob/main/Module2-ImageProcessing/M2C_Tracking_Particles.ipynb. This tutorial covers material regarding finding and tracking sub-cellular particles as described in section 4.
• **Tutorial on Stoichiometries, Propensities, and ODE Models**—https://github.com/MunskyGroup/uqbio2024/blob/main/Module4-ModelingBiochemicalReactions/M4A_Stoichiometries_Propensities_and_ODE_Models.ipynb. This tutorial demonstrates how to define stoichiometry matrices and propensity functions (section 5.1) and formulating and solving ODEs to describe biochemical reaction processes (section 5.3.1).
• **Tutorial on Stochastic Simulation Algorithm**—https://github.com/MunskyGroup/uqbio2024/blob/main/Module4-ModelingBiochemicalReactions/M4B_Stochastic_Simulation_Algorithm.ipynb. This tutorial covers how to run and analyze the results of stochastic simulations as described in section 4.
• **Tutorial on Chemical Master equation and Finite State Projection**—https://github.com/MunskyGroup/uqbio2024/blob/main/Module4-ModelingBiochemicalReactions/M4C_Chemical_Master_Equation.ipynb. This tutorial covers how to define and solve the CME using the FSP approach as described in section 5.5.1.
• **Tutorial on Bayesian Thinking**—https://github.com/MunskyGroup/uqbio2024/blob/main/Module5-BayesianThinkingAndMCMC/M5A_Bayesian_Thinking.ipynb. This tutorial provides an introduction to Bayesian analysis as described in section 6.
• **Tutorial on Markov Chain Monte Carlo**—https://github.com/MunskyGroup/uqbio2024/blob/main/Module5-BayesianThinkingAndMCMC/M5B_Markov_Chain_Monte_Carlo.ipynb. This tutorial covers how to set up and analyze problems using MCMC approaches as described in section 6.2.

### List of recorded video lectures and tutorials

• **Lecture by Eric Ron on Labeling and Imaging Technology**—https://doi.org/10.25675/10217/240614. This recorded lecture covers some of the experimental techniques discussed in section 3.
• **Tutorial by Luis Aguilera on Image Processing 1 (Basics)**—https://doi.org/10.25675/10217/240613. This recorded lecture goes through the Python tutorial for Basic image Processing discussed in section 4.1.
• **Tutorial by Luis Aguilera on Image Processing 2 (Segmentation/Tracking)**—https://doi.org/10.25675/10217/240616. This recorded lecture goes through the Python tutorials for image segmentation and particle tracking discussed in section 4.3.
• **Tutorial by Connor King on Stoichiometries, Propensity Functions, and ODE Models**—https://doi.org/10.25675/10217/240615. This recorded lecture goes over the Python tutorial for stoichiometries and propensity functions from section 5.1 and shows how to use these to define and solve ODE models of gene expression as discussed in section 5.3.1.
• **Tutorial by Alex David and Jack Forman on Stochastic Simulations**—https://doi.org/10.25675/10217/240617. This recorded lecture goes through the Python tutorial on the Stochastic Simulation Algorithm as discussed in section 5.4.1
• **Tutorial by Brian Munsky on Chemical Master Equations and Finite State Projection analyses**—https://doi.org/10.25675/10217/240612. This recorded lecture goes through the Python tutorial on Chemical Master Equations and the Finite State Projection approach as discussed in section 5.5.1.
• **Tutorial by Kaan Öcal on Bayesian Analyses and MCMC**—https://doi.org/10.25675/10217/240602. This recorded lecture goes through the tutorial on Bayesian analyses and MCMC methods as discussed in section 6.

### Drug discovery challenge repository

• **Drug Discovery Challenge**—GitHub repository: (https://github.com/luisub/qbio_paper). Zenodo: (https://doi.org/10.5281/zenodo.15376952). This repository provides codes to generate data for the course challenge.
